# Biomechanical analysis of plate systems for proximal humerus fractures: a systematic literature review

**DOI:** 10.1186/s12938-018-0479-3

**Published:** 2018-04-27

**Authors:** Ali Jabran, Chris Peach, Lei Ren

**Affiliations:** 1 School of Mechanical, Aerospace and Civil Engineering, University of Manchester, Manchester, UK; 2 Department of Shoulder and Elbow Surgery, University Hospital of South Manchester, Manchester, UK

**Keywords:** Proximal humerus fractures, Locking plates, Open reduction internal fixation, Biomechanical testing

## Abstract

**Background:**

Proximal humerus fractures are the third most common in the human body but their management remains controversial. Open reduction and internal fixation with plates is one of the leading modes of operative treatment for these fractures. The development of technologies and techniques for these plates, during the recent decades, promise a bright future for their clinical use. A comprehensive review of in vitro biomechanical studies is needed for the comparison of plates’ mechanical performance and the testing methodologies. This will not only guide clinicians with plate selection but also with the design of future in vitro biomechanical studies. This review was aimed to systematically categorise and review the in vitro biomechanical studies of these plates based on their protocols and discuss their results. The technologies and techniques investigated in these studies were categorised and compared to reach a census where possible.

**Methods and results:**

Web of Science and Scopus database search yielded 62 studies. Out of these, 51 performed axial loading, torsion, bending and/or combined bending and axial loading while 11 simulated complex glenohumeral movements by using tendons. Loading conditions and set-up, failure criteria and performance parameters, as well as results for each study, were reviewed. Only two studies tested four-part fracture model while the rest investigated two- and three-part fractures. In ten studies, synthetic humeri were tested instead of cadaveric ones. In addition to load–displacement data, three-dimensional motion analysis systems, digital image correlation and acoustic emission testing have been used for measurement.

**Conclusions:**

Overall, PHILOS was the most tested plate and locking plates demonstrated better mechanical performance than non-locking ones. Conflicting results have been published for their comparison with non-locking blade plates and polyaxial locking screws. Augmentation with cement [calcium phosphate or poly(methyl methacrylate)] or allografts (fibular and femoral head) was found to improve bone-plate constructs’ mechanical performance. Controversy still lies over the use of rigid and semi-rigid implants and the insertion of inferomedial screws for calcar region support. This review will guide the design of in vitro and in silico biomechanical tests and also supplement the study of clinical literature.

**Electronic supplementary material:**

The online version of this article (10.1186/s12938-018-0479-3) contains supplementary material, which is available to authorized users.

## Background

Fractures of the proximal humerus account for 4–5% of all fractures, making them a common upper extremity injury [1]. In the over-65 patient population, this figure is reported much higher, at 10%, often related to factors such as osteoporosis [2]. Circa 85% of the cases can be treated with a non-operative approach while the remaining complicated fractures require surgical treatment [3, 4]. The latter cases have been addressed with varying success using a variety of techniques such as K-wire fixation [5, 6], intramedullary nailing [7, 8] and open reduction internal fixation using proximal humerus plates (PHPs) [9, 10]. In terms of their surgical use, intramedullary humeral nails are advocated as they can be inserted through less invasive approaches [11]. In contrast, plate and screws demand extensive soft tissue dissection which can result in avascular necrosis [12–14]. Intramedullary humeral nails implantation, however, does require dissection of the rotator cuff tendons, which can lead to shoulder pain and complications such as the cause rotator cuff tears [15]. In terms of post-operative performance, both the PHPs and the nail implants are associated with complications such as secondary glenohumeral penetration of screws and screw loosening and pull-out [11, 16–18]. As far as the in vitro mechanical literature is concerned, the development of locking technology has had a major impact on the mechanical performance of PHPs and it promises an opportunity to minimise the aforementioned complications. However, a gap still exists between in vitro results and the clinical outcomes as several in vivo reports describe high incidence of complications such as screw penetration of the articular surface [12, 19–21] and sub-acromial impingement of the locking plate [6, 22, 23]. In elderly patients, these complications are worse and stable fixation is even harder to achieve due to the poor anchorage of screws to the osteoporotic bone.

During the last two decades, a series of new PHPs have been developed, based on different design philosophies. Several in vitro biomechanical comparisons of PHPs have been conducted with the aim of not only comparing their in vitro properties of the plates but also the technologies and techniques associated with them.

One of the approaches to enhance the in vivo functionality of an implant is to optimise its design. This is because the design processes derived from this approach often involve the in vitro testing of the proposed designs before the in vivo trials. To be specific, evaluation of the in vitro studies should include the implant’s performance and the experimental protocols used.

It should be noted that the term protocol here includes many aspects, noteworthy of which are four: loading conditions, methods of applying the loads, criteria set to define implant’s failure (failure criteria), and the parameters determined to indicate the implant’s performance. An ideal protocol would be both standardised and reproducible, consisting of loading conditions, methods, and failure criteria that all fully depict the in vivo scenario. Also, the parameters determined in an ideal protocol would be strong, quantitative indicators of the implant’s in vivo functionality.

The majority of the literature on PHPs consists of in vivo clinical studies (e.g. clinical trials, observational studies, and case studies) and most of the literature reviews are also limited to them. Comprehensive reviews of the in vitro biomechanical studies are noticeably scarce. The few that do exist have put more emphasis on studies’ results instead of the protocols used. Also, they often reviewed biomechanical studies not as the primary aim but as a part of a broad review of all types of studies (including clinical studies). Furthermore, the inclusion criteria that they set are strict, allowing only a specific group of studies with certain types of PHPs and fracture patterns, making it difficult to draw generalised conclusions.

Thus, a review of the in vitro biomechanical studies is needed as it would not only allow the design of further in vitro biomechanical studies but also the comparison of the performance of different plates. The latter will be of particular importance to both the clinicians with the clinical decision making and for engineers with the design process of better implants. Also, in vitro biomechanical studies of PHPs currently lack standardisation. For example, a census is required on the choice of clinically important parameters but their sheer number makes the comparisons of the plates’ performances very challenging. Conducting a literature review will help achieve this.

To address the aforementioned shortcomings in the current literature, a literature review was conducted to answer three research questions:

1. What is the state of the art of in vitro experimental testing to assess the biomechanical performances of proximal humerus plates?
2. What are the controversial issues in the plate-based treatment of proximal humerus fractures revealed by previous experimental studies?
3. How would the in vitro biomechanical testing help to address those issues?

The studies were categorised thematically according to the technologies and techniques investigated in them before comparison so that a census could be achieved for each category where possible. A thorough review of the protocols will assist the design of future studies which are more close to the ideal and provide better insight into the issue of standardisation. It is also hoped that the seemingly challenging task of comparing the in vitro and in vivo functionality of plates will be simplified if this review is studied alongside with reviews of the clinical literature.

## Survey methodology

A systematic electronic search of Web of Science, Scopus, MEDLINE (via PubMed) and Google Scholar database was conducted by A. J. and L. R. from the earliest available until December 2017 in each database, using the search criteria:

(“proximal humer*” OR “shoulder”) AND (“fracture*”) AND (“fixation” OR “php” OR “angle stable” OR “lock* plate” OR “blade plate”) AND (“*mechani*”)

Only the studies that performed in vitro biomechanical testing of PHPs and were written in English and had published in a peer-reviewed journal were included. Literature reviews, clinical trials, observational and case studies were excluded.

**Fig. 1:**
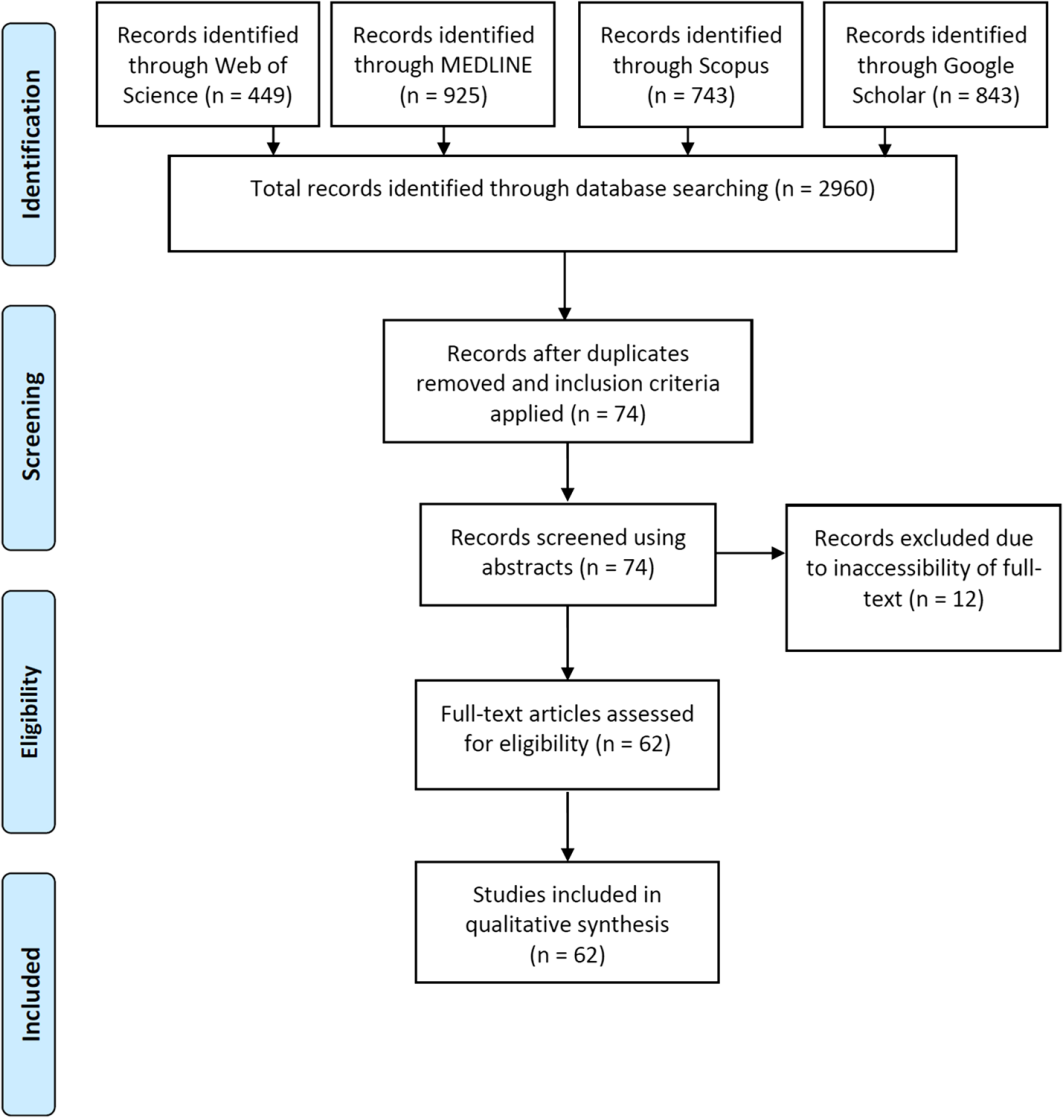
Literature search profile

The search yielded 2960 hits (Fig. 1). Titles and abstracts of the obtained studies were examined to determine their eligibility. After removing duplicates and applying the inclusion criteria, only 74 were found to be relevant. For twelve of these 74 studies, full-text was inaccessible and the abstracts alone did not provide sufficient information to allow for adequate reviewing. Remaining 62 studies were therefore included in the review, details of which have been summarised in Additional file 1: Table S1.

The earliest study was by Koval et al. [7] in 1996 and the latest being by Hsiao et al. [24] in 2017. From the nature of literature, one could categorise the included studies on a variety of bases such as the type of plates tested, types of parameters determined and even chronologically. Here, since our focus lied on biomechanical testing, categorisation was according to the type of loading performed.

**Fig. 2:**
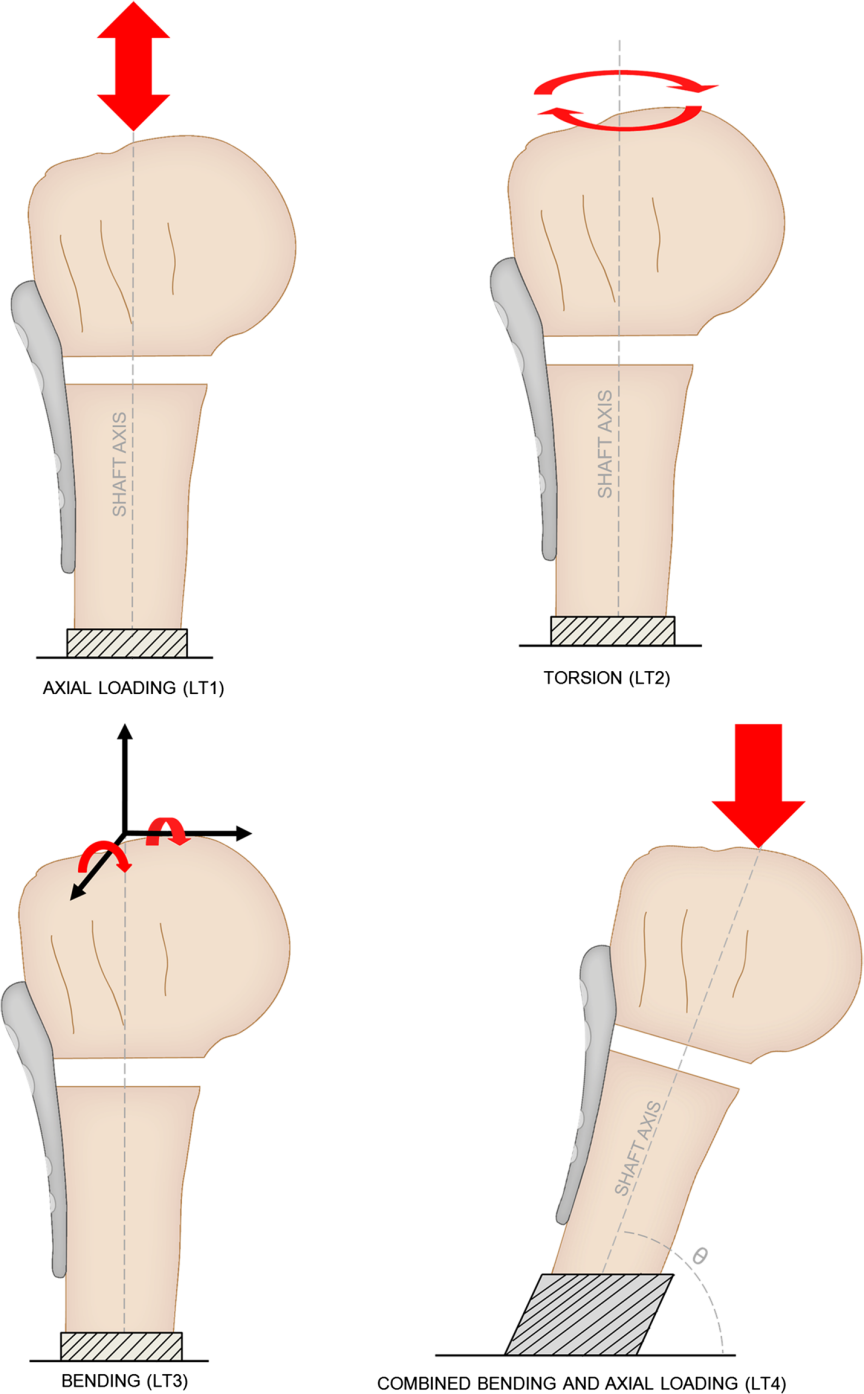
Four types of loading performed in humerus-only testing studies

Most studies (n = 51) employed relatively simple forms of mechanical testing: axial loading, torsion or bending moment, applied directly on the humerus. They formed the “humerus-only testing” category as they involved humeri specimens with tendons and musculature removed. Accordingly, they were further divided into four subcategories: axial loading (LT1), torsion (LT2), bending (LT3) and combined bending and axial loading (LT4) as illustrated in Fig. 2.

Most humerus-only studies (26) involved only one type of loading, but in others, combinations of two or three were performed (Table 1). This further complicated the comparison of their results because very often, the same specimen within a single study underwent several loading types, making it difficult to isolate the effects of each loading type. To address this, each study’s order of loading was carefully studied and rationale was provided, where possible, for their inclusion or exclusion into the corresponding subcategory.

**Table 1:** Loading types for humerus-only testing and the number of studies in which they were performed

Loading type/s (LT)	Description	Number of studies	References
1	Axial loading	7	[24–30]
2	Torsion	2	[31, 32]
3	Bending	3	[33–35]
4	Combined bending and axial loading	14	[7, 36–48]
1 + 2		4	[49–52]
2 + 3		10	[8, 9, 53–60]
2 + 4		2	[61, 62]
3 + 4		1	[63]
1 + 2 + 3		2	[64, 65]
1 + 2 + 4		5	[66–70]
2 + 3 + 4		1	[10]

**Fig. 3:**
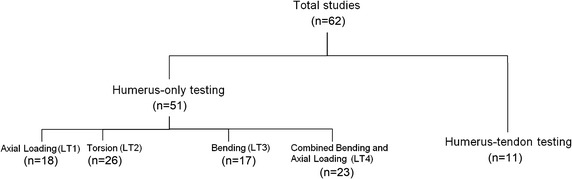
Overall categorisation of studies included in the literature review

Other than the humerus-only studies, the remaining eleven studies performed more indirect loading of the humerus, with the use of tendons to achieve complex movements. Thus, they were collectively named “humerus-tendon” testing group (Fig. 3).

## Biomechanical testing of proximal humerus plates

Overall, the most common loading type was torsion LT2, followed by LT4, LT1 and LT3. For each of the four mechanically simple loading type categories (LT1-4), both cadaveric and synthetic humeri had been tested. Each category also included studies testing two- and three-part fracture models as well as static and cyclic loading. Overall, synthetic humeri were assessed in ten studies [41, 47, 55, 59, 62, 66, 70–73] while others tested human cadaveric humeri. Only two studies involved four-part fractures [72, 73], both of which belonged to humerus-tendon category.

### Loading type 1: Axial compression and tension

#### Loading conditions

The LT1 involved the mechanically simple loading of the humerus along its shaft axis. In most studies, this was axial compression, but Instrum et al. [25] imposed tension to simulate the longitudinal distraction of humerus caused by the upper limb weight. Chudik et al. [36] did perform axial compression but only on unplated humeri during the preloading stage of their study, while the main focus was LT4. Thus, their study was not included in this category.

For Dietz et al. [52], static LT1 and cyclic LT2 were applied simultaneously, and vice versa, while for Schumer et al. [51], only static LT1 and cyclic LT2 loads were simultaneously applied. The most common set-up was to fix the humeral shaft and load the humeral head, which was often potted in a polymer holder. In static tests, displacement-control loading at a rate of 5 mm/min have been most frequently employed [24, 26, 27, 30, 49, 66, 67, 70] while displacement rate of 0.1 mm/s [68, 69] and 20 mm/min [25] and load rates of 1 N/s [28] and 20 N/s [51] have also been used. In terms of the loading order, seven [24, 25, 27, 28, 51, 68, 69] of the eleven studies involving both static and cyclic axial loading, performed a static loading-to-failure step at the end to characterise constructs’ load to failure behaviour.

Failure was most often defined as the complete (or irreversible) closure of fracture gap [24, 30, 50–52, 68, 69] and as the clear deviation in linearity of the load–displacement curve [26, 27, 50, 52]. Based on the load–displacement curve plots, failure was also defined as a point of a major drop in the load [24, 51] and this was elaborated by Zettl et al. [28] to be a greater than 30% drop in the pressure. Another criterion described failure as humeral displacement greater than 20 [29] or 30 mm [28] on the load–displacement curve.

#### Measurements and data analysis

For quantitative analysis, most studies recorded the universal testing machine’s actuator loads and displacements. In five studies [49, 64, 65, 68, 69], relative movements of the proximal and distal fracture fragments were recorded during tests using optical and ultrasound-based three-dimensional (3D) motion analysis systems. This was often achieved with the use of reflective markers attached on either side of the fracture gap to describe movements in terms of translations and rotations in the x-, y- and z-axes.

Linear elastic stiffness of the construct, i.e. the gradient of the linear elastic region of the load–displacement curve was most commonly calculated to compare mechanical performance. At the start of their tests, Dietz et al. [52] loaded humeri under elastic conditions to calculate their initial stiffness. After introducing the fracture and fixating the implant, they tested the same humeri to find their second stiffness. They then reported the difference between these two stiffness values as the “loss of stiffness” which was represented as a percentage. Load to failure was also found from load–displacement data, often in studies with initial submaximal cyclic loading and final static loading to failure tests. Moreover, displacement at failure [28], maximum load [30] and yield load [25] was also reported in the literature. The latter was defined graphically as the peak of the load–displacement curves and in case of Instrum et al. [25], it was the tensile yield load. For cyclic loading, number of cycles to failure [26, 51], plastic deformation after a certain number of cycles [27, 28, 50] and maximum [39] and final [51] plastic deformation have been calculated. Hsiao et al. [24] determined peak-to-peak (inter- cyclic) displacement and cumulated deformation at specific cycles.

### Loading type 2: Torsion

#### Loading conditions

LT2, torsional moment on the humerus along the shaft axis, was the most prevalent type of loading in literature. The most popular setup was the direct application of torsion using a material testing machine on a holder (e.g. polymer pot) which held the humerus, with the distal fragment fixed. Three studies [54, 55, 60] imposed torsion on the distal fragment instead of the humeral head. Indirect loading has also been achieved via the use of cables connected to a holding construct [8, 62] and by projecting devices connected parallel [9, 53], and perpendicular [56], to the shaft axis. Internal and external rotations have been performed both in separation and union, from which different parameters and criteria were determined to define the behaviour of bone-plate constructs.

In general, for both static and cyclic loading, the studies could be separated according to the ascending order of their angular displacement rates: 1°/s [10, 59, 61, 66], 5°/min [67], 0.1°/s [68, 69], 0.5°/s [9, 31, 32, 53, 57] and 20°/s [60] or the displacement rates: 1 mm/min [8], 5 mm/min [62] and 12 mm/min [70]. Similarly, large varieties were found among the values and ranges of torques, angles and the time duration of the tests. In case of Foruria et al. [32], rotational moments created by the subscapularis and infraspinatus muscles during shoulder elevation were simulated, based on a previous biomechanical study [74].

Although the studies involving torsion tests to failure were common, for most studies, separate failure criterion was not proposed for the torsion tests. From those that did, Unger et al. [58] set it to be a torsion greater than 4° during one load cycle while for Roderer et al. [57], it was axial displacement greater than 30°.

#### Measurements and data analysis

In terms of measurements, most studies measured angular displacement from actuator as well as the actuator load but interfragmentary motion was also recorded by nine studies [32, 49, 56–58, 61, 64, 68, 69] using 3D motion analysis systems. In addition to torsional stiffness, loss of stiffness [8, 52] after a set number of load cycles has also been calculated. Huff et al. [59] computed the peak torque of the first and the last cycles in the internal and external rotation. Other parameters to be reported were torque-at-failure, angular displacement-at-failure, maximum torque, angular displacement at maximum torsion and energy at failure (area under the torque-displacement plot) [32].

### Loading type 3: Bending

#### Loading conditions

**Fig. 4:**
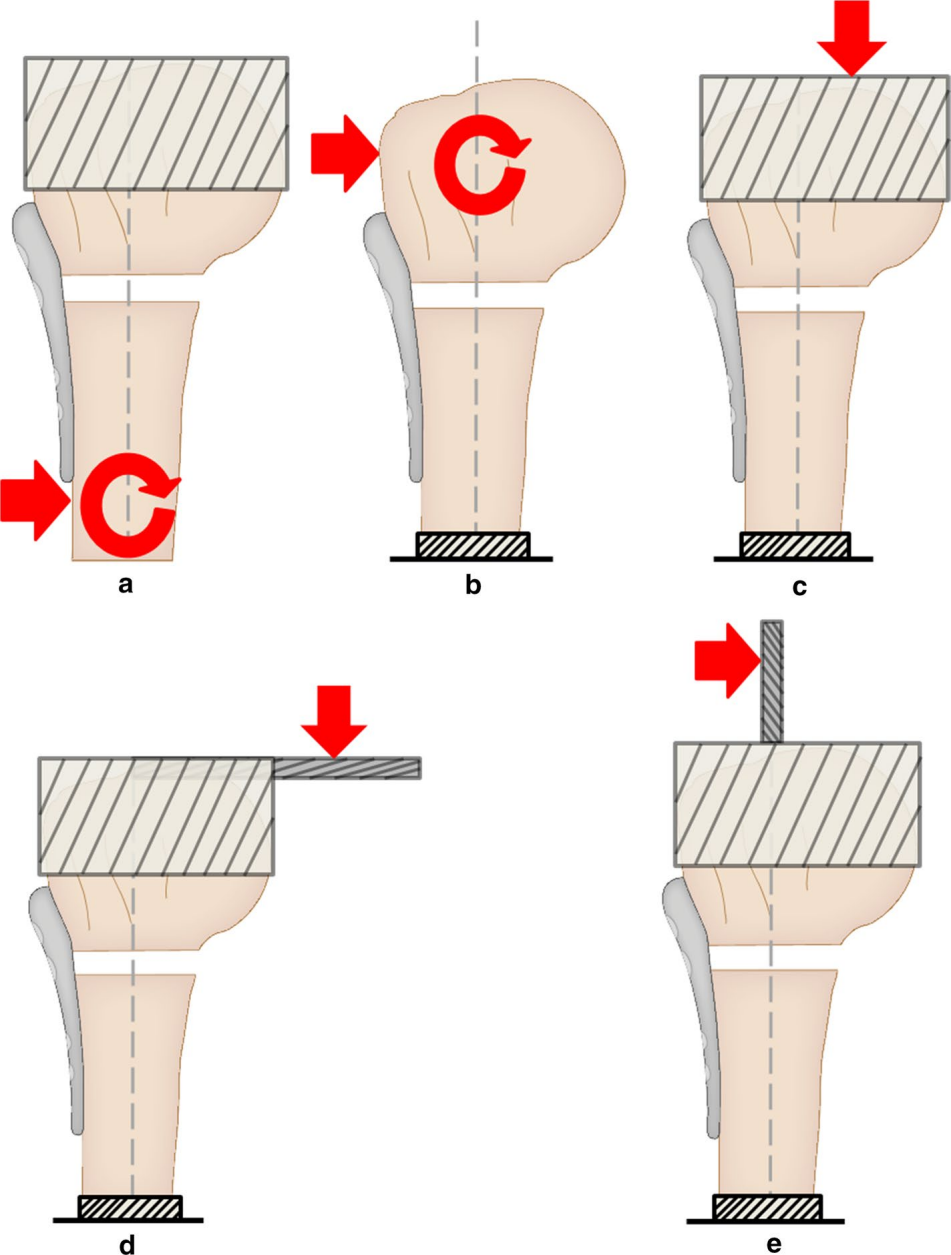
Five common experimental setups used in literature for applying bending loads. **a** Direct shaft loading. **b** Direct head loading. **c** Eccentric loading without rod. **d** Eccentric loading with horizontal rod. **e** Eccentric loading with vertical rod

Loading type 3 (LT3) was the bending of the humerus, commonly by loads along either of the two axes perpendicular to its shaft axis (Fig. 2), resulting in either an extension/flexion or varus/valgus moment. In terms of the protocol, Chow et al. [34] and Weeks et al. [35], Lill et al. [64] and Duda et al. [65], and Ruch et al. [53] and Kitson et al. [9] were very similar. Eight studies [8, 33–35, 54, 55, 59, 60] subjected humeral shafts to perpendicular loads (Fig. 4A), in a cantilever fashion, with the humeral head fixed. To achieve the required head fixation, either an embedding material such as a resin [33–35, 59, 60], a low-melting point metallic alloy [55] or hard gypsum [8] was used, or, in case of Edwards et al., the head was held by a custom-made bone holder consisting of a tube and spiked screws [54]. All of these studies conducted varus bending by orthogonally loading the shaft along the frontal plane. Huff et al. [59] applied valgus, extension and flexion bending in addition to varus.

Several rationales were presented for the loading conditions used in these eight studies. In case of Mathison et al., the load was transmitted 70 mm distal to the third most proximal row of plate’s screw holes with the aim of replicating rotator cuff’s moment during abduction [33]. Most of the other seven studies aimed to load the humeral shaft such as to achieve a bending moment of 0–7.5 Nm at the fracture site [8, 34, 35, 54, 55, 60]. Chow et al. [34] and Weeks et al. [35] performed this on the basis of a biomechanical study by Poppen and Walker [75]. They aimed to replicate the supraspinatus forces on bone-plate constructs during the early stages of healing under shoulder immobilisation support. Mechanically, this loading is comparable to humeral immobilisation followed by a varus force acting directly at the supraspinatus insertion site.

In other eight studies, humeral head was loaded and the shaft fixed [9, 10, 53, 56–58, 63–65]. Roderer et al., Lever et al. and Kralinger et al. achieved this by fixing the humeral shaft and directly loading the humeral head in the desired direction (Fig. 4b) via a biaxial material testing machine or a 3D spinal loading simulator [56, 57, 63]. Lever et al. loaded the humeral head in the posterior direction for flexion and in a medial direction for abduction [63]. Four studies involved attachment of a circular plate (Fig. 4c) and/or a long metal rod projected horizontally (Fig. 4d) to the humeral head [10, 56, 64, 65]. The load was applied to the plate or the rod, at an offset distance away from the shaft axis, using a vertical machine actuator. This offset point was set along different directions to produce extension, flexion, valgus and varus bending to the constructs. Contrarily, Kitson et al. and Ruch et al. fixed a metal rod that projected vertically (Fig. 4e), along the shaft axis, and loaded it perpendicularly at a set height above the tip of the humeral head [9, 53]. Four of these eight studies performed all of the four key humeral bending movements: extension, flexion, varus, and valgus [9, 10, 53, 57].

Roderer et al. tried to replicate the peak resultant moment during several activities of daily livings such as combing, setting down a 2 kg weight on a board at head height and holding a 10 kg weight, developing on the findings of a previous biomechanical study by Bergmann et al. [76]. Kralinger et al. applied varus bending to reproduce the pull of the supraspinatus and medial shearing (lateral displacement of the head) to simulate the pull of the pectoralis major [56]. Lill et al. and Duda et al. only performed varus bending as the former aimed to reproduce the in vivo displacement of the fracture which occurs mainly due to the tension of the supraspinatus tendon [64, 65]. The study by Unger et al. was unique in the LT3 category in the sense that it neither involved the application of cantilever loads on the shaft nor was the humeral head loaded [58]. Instead, humerus was loaded on the shaft to produce varus bending, with the humeral head set in a custom holder which was connected to a ball-socket joint.

Unger et al. defined failure to be the increase of angular tilting of over 0.5° in varus within 100 load cycles at the lower load magnitudes. Moreover, failure criteria based on the varus collapse and passage of 25,000 cycles during the cyclic tests were implemented by Chow et al. and Weeks et al.

#### Measurements and data analysis

3D motion analysis systems were used to monitor the relative movements of the fracture fragments [56–58, 64, 65]. Mathison et al. [33] used digital image correlation to not only find the relative movement of fracture surfaces but also the local strain across the surface of the specimen. To achieve this, speckling pattern was applied to the specimen surface before starting the tests, which acted as the reference point. During the course of loading, photographs of the specimen were taken which allowed the computation of the relative displacement of the speckles due to the load translations.

Force–displacement data was generally used to measure elastic stiffness and failure load of the bone-plate construct for the corresponding type of bending. For cyclic tests, load per cycle [64, 65] and the mean displacement per cycle [34, 35] and its inverse (number of cycles required to achieve one millimetre of displacement) [34, 35] have been determined. Other parameters calculated for the comparison of constructs’ performance include the displacement and number of cycles at a set interval and to failure as well as the difference in the peak load of the first and last load cycle [59].

### Loading type 4: Combined bending and axial loading

#### Loading conditions

Twenty-three studies conducted LT4 all of which loaded the humeral head except Kwon et al. [61] who loaded the shaft instead. Koval et al. fixed the humeral shaft at 20° of abduction to simulate the primarily shear loading (approximately twice the amount of shear than compression) of the bone-plate construct. This set up acted as the basis for nine biomechanical studies [37, 41, 42, 46, 47, 62, 63, 66, 70]. As well as 20° abduction, Lever et al. [63] mounted the shaft at 20° of forward flexion in a similar manner to Koval et al.

Poppen and Walker computed the force vectors at the glenohumeral joint during isometric scapular plane abduction [75]. Inspired by this study, Hymes et al. and Sanders et al. applied vertical loads to the humeral head 30° posteromedial to the anteroposterior in the plane of rotator cuff pull. This represented the glenohumeral joint force in 0° of abduction that occurs at the surgical neck due to rotator cuff [10, 40]. Similarly, Burke et al. imposed a vertical load of 532.6 N on the head [41]. This was to simulate the maximum reaction forces in the shoulder of a 72 kg average man at 90º of isometric scapular plane abduction, adapted from the Poppen and Walker study [75]. Kwon et al. loaded the humeral shaft with the head fixed and the scapulothoracic motion absent such that the rotation of the specimen from 30° to 80° approximately recreated the glenohumeral rotation that occurs through 30° to 120° shoulder abduction [61]. The 20-50% body weight joint compressive load applied during this cyclic abduction simulated in vivo joint compressive forces described by Poppen and Walker [75].

Six studies based their loading conditions on one of the two studies by Bergmann et al. [76, 77] to introduce glenohumeral contact forces measured in vivo during activities of daily living [39, 43–45, 68, 78]. Out of these, four studies [39, 43–45] fixed humeri in lateral angulation to perform varus movement, where Roderer et al. [45] and Schliemann et al. [43, 48] tilted the shaft at an angle of 25° while Gradl et al. [39] oriented them at 20°. The remaining two studies were by Katthagen et al. [68, 69] where loads were transmitted vertically to the humeral head with the shaft inclined at 20° in adduction, developed from the studies by Bergmann et al. [76] and Westerhoff et al. [79]. As an attempt to evenly load the specimens, Roderer et al. [45] and Gradl et al. [39] used a polymethyl methacrylate load-cup shaped as negative of the humeral head to represent the glenoid. The former also prevented relative rotation between the cup and the humeral head by applying sandpaper strips on parts of the cup that was in contact with the head.

Erhardt et al. loaded the humeral head while the humeral shaft was set at 30° flexion and 30° abduction to simulate the physiological load vector of a shoulder with an intact rotator cuff during 30°–90° abduction [38]. This load vector is perpendicular to the glenoid plane and generates a glenohumeral contact force of 240 N at 30° of abduction and increases up to 582 N at 90° abduction, as defined by Konrad et al. [80].

Ponce et al. [37] set separate criteria for the comminuted and non-comminuted specimen. For the former, it was the closure of the medial cortical defect while for the latter, it failure load was the maximum load recorded. Specimen angular displacement of 15° in an unloaded condition was considered by Gradl et al. [39] to be failure. Similarly, Roderer et al. [44] and Schliemann et al. [43, 48] defined failure as an increase of varus angular tilting greater than 0.5° within 100 load cycles at lower magnitude (constant 15 N), determined from the data from the 3D motion analysis system. In another study, Roderer et al. [45] employed a criterion of humeral head migration greater than 2 mm, based on fluoroscopic assessment.

#### Measurements and data analysis

Six studies [41, 43, 44, 48, 61, 68, 69] utilised 3D motion analysis systems for the measurement of humeral and interfragmentary motion. Roderer et al. [44] and Schliemann et al. [43, 48] also recorded the relative motion between the humerus and the plate. Other direct measurements taken were the number, amplitude and distribution of microcracks formed on humeri during testing, which was made possible via the use of acoustic emission testing by Hymes et al. [40]. Fluoroscopic assessment which is often conducted for qualitative analysis was used by Roderer et al. [45] to track the migration of humeral head after a certain number of load cycles from the recording of the relative position of radiopaque reference points with respect to the implant. Bulut et al. [47] measured displacements between fracture ends with a camera and extensometers to allow calculation of the gauge length elongation.

Parameters such as stiffness, ultimate load, as well as load and energy to failure, based on the load–displacement data acquired, have been of principal interest in most studies. In studies performing cyclic tests, the number of cycles at failure and the displacement at a given cycle number have been recorded. Inspired by the work of Poppen and Walker [75], Chudik et al. recorded displacement at 0.3 and 0.6 kN specifically to represent the forces on the humeral articular surface through the humeral geometrical centre during 30° and 90° arm abduction respectively.

Using the acoustic emission testing, Hymes et al. [40] located and recorded the number of the microcracks that were either theoretically locatable (type I) or not (type II). By combining the location information of these microcracks and the X-ray data, damage propagation was visualised in real time. From this, they plotted the number of each crack type against the number of cycles.

### Complex loading using humerus-tendon setup

#### Loading conditions

Unlike the previous four types of loading, these studies involved tests that were both complex and physiologically more accurate. These eleven studies could be further divided according to the type of tendons used in them: cadaveric [81–86] or synthetic [71–73, 78, 87].

From the six studies testing cadaveric tendons, two studies by Voigt et al. involved the use of a RASS (robot-assisted shoulder simulator) along with hydraulic systems to control the pull of supraspinatus, subscapularis and infraspinatus and teres minor via brass wires sutured to the respective muscles [82, 83]. Both studies replicated the rotator cuff tension during glenohumeral elevation while one also recreated the axial loading at 0° and 60° of glenohumeral abduction as well as the external rotation at 0° abduction with the load magnitudes taken from previous in vitro biomechanical studies [88, 89]. Rose et al. [90] mimicked 10°–60° cyclic abduction by loading the supraspinatus, subscapularis and infraspinatus muscles for 5000 cycles or to failure, with 2.75 kg of mass affixed to distal humerus in order to approximate the mass of the upper extremity. The same three muscles were loaded by Walsh et al. [81] to represent glenohumeral abduction of 30°. Two studies [85, 86] testing cadaveric tendons were based on the biomechanical study by Osterhoff et al. [71]. Sinatra et al. [85] used custom-made shoulder testing setup connected to a material testing machine to recreate 50–100° single plane shoulder abduction. This was achieved with the application of cyclic tensile forces to supraspinatus, infraspinatus, subscapularis, and teres minor tendons while lifting 5 lbs to simulate arm weight. Similarly, Arvesen et al. used custom-made shoulder testing setup to perform 35–65° active glenohumeral abduction. To achieve this, cyclic tensile loads were applied to supraspinatus, subscapularis, and teres minor tendons.

The remaining five studies used different materials as synthetic tendons and all performed glenohumeral abduction. Both Brunner et al. [78] and Kathrein et al. [87] used shoulder joint test bench to perform abduction along the scapula plane and 15°–45° adduction. Pneumatic muscles mimicking the supraspinatus and deltoid for abduction and pectoralis major and teres major for adduction were attached to the insertions of the respective muscles using webbing straps. In case of Brunner et al., the applied muscle forces were comparable to those calculated in a finite element study by Terrier et al. [91]. Da Graca et al. simulated infraspinatus tendons for supraspinatus and subscapularis tendons as well as axillary recess, using leather straps. Straps were glued to the insertion points of the corresponding tendons at one end while on the other end they were drilled into an aluminium scapula that had holes for the supraspinatus, infraspinatus and subscapular fossae. Using this custom-made setup, abduction and internal rotation to failure were carried out.

In a similar fashion, Osterhoff et al. [71] used polyester webbings to represent the pull of muscles and attached them to the corresponding insertions using a cyanoacrylate adhesive for tendon-bone fixation. Pull of supraspinatus and deltoid tendon was replicated for the abduction of 45° to 60° while lifting a 3.75 kg weight at the distal humerus. Also, to simulate the action of infraspinatus/teres minor and subscapularis, constant loads of 25 N each were applied. Similar to da Graca et al., the loading by Osterhoff et al. was cyclic, albeit lasting only 400 cycles as opposed to until failure. Clavert et al. used a custom-made testing setup connected to a mechanical testing machine and used polyethene rope glued to superior and lateral greater tuberosity aspects to simulate 0° glenohumeral abduction and neutral rotation, relative to the scapula plane or 90° of abduction in the scapular plane.

In general, fracture criteria were not explicitly stated in these studies, presumably due to the fact that the loading range of motion was already well-defined in terms of maximum and minimum magnitudes, deeming it unnecessary to set additional criterion. da Graca et al. who defined failure as the sudden drop in the load applied by the universal testing machine, was among the exceptions.

#### Measurements and data analysis

Force–displacement data was often used to calculate the load and displacements at failure or at a specific number of cycles. Kathrein et al. [87], similar to Brunner et al. [78], reported the maximum resulting forces on the glenoid and of the individual muscles. Voigt et al. [83] recorded the deltoid forces necessary to elevate the arm in set positions, and determined the efficiency of supraspinatus as well as the ratio of deltoid force to arm elevation angle (N/°) in different phases of elevation.

With the aid of 3D motion analysis system, Kathrein et al. [87] recorded the relative motion of the humeral head and the plate and the change at the minimum value of abduction (varus impaction) for each load cycle. Brunner et al. [78] used a 3D motion analysis system, fracture gap motion along the shaft axis and the maximum varus tilt of the humeral head was recorded for each load cycle.

Osterhoff et al. utilised inductive sensor system to record fracture gap distance during the tests. Based on this data, they determined the intercyclic motion at a set number of cycles as well as the fragment migration and the change in the fracture gap distance. Arvesen et al. [86] used video recorder to record fracture gap distance and calculated intercyclic change in fracture gap. Brunner et al. [78] performed X-ray scans before testing and after every 500 cycles to determine the changes in the length of each telescoping pin of the Humerus Block implant, as well as the distance between the pins’ tips and the humeral head cortex.

## Comparison of plate technologies and techniques

The basis for most studies has been to investigate the technologies and techniques related to plate-based management of proximal humerus fractures. These include the investigation of locking and non-locking screw technology, polyaxial and monoaxial locking screws, rigid and semi-rigid implants, the importance of calcar region and cement augmentation of the humerus. It should be noted that PHPs have been biomechanically compared with several other non-plate treatments, the most common of which is the intramedullary nail [7–10, 32, 42, 50, 52–54, 56, 64, 73]. The focus of this review, however, is the plate-based fixation so the results pertaining to other treatments will not be discussed here. As for the plates, a wide range of locking and non-locking plates were tested (Table 2), among which, PHILOS plate (Synthes, Paoli, PA, USA) and its variants, were tested the most.

**Table 2:** Brief description of proximal humerus plates tested in the literature

Plate name	Manufacturer(s)	Description
Proximal humerus internal locking system (PHILOS) plate	Synthes (Paoli, PA, USA); Clinical House (Dusseldorf, NRW, Germany); Stratec (Birkenfeld, BW, Germany)	Locking plate allowing insertion of mono-axial locking screws. Relatively low cross-section thickness
AxSOS plate	Stryker (Kalamazoo, MI, USA)	Locking plate allowing insertion of mono-axial locking screws
TIFIX plate	LITOS (Ahrensburg, SH, Germany)	Locking plate allowing insertion of mono-axial locking screws
PERI-LOC plate	Smith and Nephew (Memphis, TN, USA)	Locking plate allowing insertion of mono-axial locking screws
Humeral telescoping screw (HTS) plate	M.O.R.E. Medical Solutions (Rostock, MV, Germany)	Locking plate allowing insertion of mono-axial locking screws in addition to a telescoping screw
Non-contact bridging (NCB) plate	Zimmer (Warsaw, IN, USA)	Locking plate employing polyaxial screws, instead of the common monoaxial screws
DiPhos-H plate	Lima Corporate (San Daniele del Friuli, UD, Italy)	Locking plate manufactured from PEEK (poly-ether-ether-ketone), allowing insertion of mono-axial locking screws
PEEKPower plate	Arthrex (Naples, FL, USA)	Locking plate manufactured from PEEK allowing insertion of mono-axial locking screws
Spatial subchondral support (S3) plate	Depuy (Warsaw, IN, USA)	Locking plate allowing insertion of mono-axial locking screws. Placed more distally on the humeral head to reduce risk of subacromial impingement. Also allows insertion of smooth pegs and threaded pegs to avoid glenohumeral penetration of screws
Humerus block	Synthes (Salzburg, Austria)	Semi-rigid locking plate with four telescoping fixation pins where proximal end of each pin has a telescoping mechanism to its shortening under load. Pin tips also include three curved springs that are intended to improve pins’ fixation in cancellous bone and prevent their perforation into the glenohumeral joint
Button fix	Synthes (Solothurn, SO, Switzerland)	Semi-rigid PEEK plate with 4 threaded holes to allow insertion of 4 Kirschner wires using an aiming device
Semitubular blade plate	Synthes (Paoli, PA, USA)	Non-locking plate with a bend at its proximal end to form a blade
90° blade plate	Zimmer (Warsaw, IN, USA)	Non-locking plate with blade oriented at 90°
AO T plate	Synthes (Paoli, PA, USA)	Non-locking plate with a T-shaped profile
Cloverleaf plate	Synthes (Paoli, PA, USA)	Non-locking plate with a wide profile in contact with the humeral head

N.B. Only products that have been explicitly named in the studies have been included

### Locking vs. non-locking screws

Development of the locking screw technology is one of the major milestones in the management of proximal humerus fractures. Locking screws have threaded heads that lock into the plate’s screw holes to create an angular stable fixation. While the conventional non-locking screws rely on the bone-plate interface for stability, locking screws are reliant on the bone-screw interface instead, resulting in theoretically lower friction [92]. The failure mode of locking plates also differs from that of conventional non-locking ones. Non-locking plates typically fail in series due to the toggling, loosening or the pulling out of the screws whereas the failure of locking plates demands simultaneous pullout or failing of all screws [93]. As a result, locking plates exhibit superior pullout strength and stiffness as these properties are related to the construct in entirety and not to individual screws [94]. This does prove advantageous for small to moderate loading range but catastrophic under high impact forces. General literature of proximal humerus fractures is laden with the use of locking plates but the most frequently experimented plate employing this technology is the PHILOS plate, which has been tested against plates such as Non-Contact Bridging plate (Zimmer,Warsaw, IN, USA) [28], humeral suture plate (Arthrex, Naples, FL, USA) [82], AO T-plate (Synthes, Paoli, PA, USA) [8, 50] and telescrew plate (M.O.R.E. Medical Solutions, Rostock, MV, Germany) [29] as well as proximal humerus nails [8, 32, 50, 52, 73].

The theoretical advantages of locking plates are supported by Seide et al. [26] and Walsh et al. [81]. The former demonstrated superior elastic stiffness and better fatigue behaviour for TIFIX locking plate (LITOS, Ahrensburg, SH, Germany) under axial compression as compared to the non-locking version of the same plate. Similarly, Walsh et al. recorded higher maximum load to failure for constructs treated with Synthes locking plate than those with non-locking cloverleaf plates in cadaveric shoulders during 30° glenohumeral abduction.

Traditional blade plates have used non-locking screws and have been tested, often as representative of the non-locking plate category. Weinstein et al. [31] showed that locking plates exhibit significantly larger stiffness than blade plates in the cyclic external rotation. Siffri et al. [55] also reported that in cadaveric specimens, in comparison to blade plate constructs, locking plate constructs had significantly greater torsional stability. Statistically similar stability, however, was recorded in cantilever bending for the two construct groups.

Kwon et al. [61] loaded humeri that had been treated with either the cloverleaf (Synthes, Paoli, PA, USA) or the blade plate (Synthes, Paoli, PA, USA), both of which were non-locking, under 30°–120° cyclic abduction and rotation and reported no significant differences between the performance of the two construct groups. Similarly, Gillespie et al. [46] loaded locking plates, standard non-locking plates and non-locking blade plate constructs in 20° of abduction and demonstrated that the blade plate constructs exhibited greater stiffness than locking plate while the locking plate was stiffer than the standard non-locking plate. These differences in the mean stiffness among the three constructs, however, were not statistically significant.

### Polyaxial vs. monoaxial locking screws

Clinical studies for locking plates, in particular, the PHILOS plate, report a significant number of complications due to the perforation of screws through the humeral head. One potential solution is to use polyaxial screws in them. This has been named the second generation locking technology as it allows the screw direction to be adjusted before locking, as opposed to the conventional locking systems where screw angles are pre-defined and therefore, monoaxial. One plate employing this strategy is the Non-Contact Bridging plate (NCB, Zimmer, Warsaw, IN, USA) biomechanical performance of which has been tested in three studies [28, 38, 57]. Zettl et al. [28] demonstrated statistically similar performance between the NCB plate and PHILOS plate under axial compression despite using fewer and thicker screws for NCB plate. However, Erhardt et al. [38] revealed that during simulated 30° flexion and 30° abduction, insertion of polyaxial screws instead of monoaxial ones had no significant effect on the perforation of screws.

### Importance of calcar region

The importance of recreation and mechanical support of the humeral medial column is emphasised in clinical literature for construct stability [95] and ergo for the reduction of complications such as screw perforation of the articular surface and varus collapse. An in vitro biomechanical study by Lescheid et al. [66] also supports this by reporting higher axial, torsional and shear stiffness with the restoration of medial cortical contact.

One approach to provide this mechanical support is by placing screws across the medial calcar region. Katthagen et al. [69] performed in vitro axial loading, torsion and bending of cadaveric humeri with two-part fractures that had been treated with PHILOS locking plate. No significant difference was detected with the insertion of calcar screws. In contrast, Zhang et al. [70] reported higher axial and shear stiffness for synthetic humeri with two-part fractures treated with medial support screws than those without medial support screws. Also, Erhardt et al. [38] achieved increased resistance to screw perforation during flexion and abduction due to the insertion of the inferomedial support screw.

A cadaveric study of three-part fractures by Ponce et al. [37] reports significantly higher mean load to failure and mean energy to failure with the use of calcar screws in PHILOS locking plate during varus collapse tests. Similarly, according to Burke et al. [41], insertion of inferomedial screws in PHILOS plate led to significantly lower mean interfragmentary motion and increased the load to failure in humeri with three-part fractures.

With the aim of providing the required mechanical support (compressive strength) to the medial column, Gardner et al. [96] proposed the use of fibular allografts in the intramedullary canal of the proximal humerus. In biomechanical studies, constructs with fibular allograft augmentation (PHP with allograft) exhibited better stability than non-augmented constructs (PHP only) under axial compression [27], varus bending [33, 34] and 45°–60° simulated glenohumeral abduction [71], with higher stiffness and failure loads. Similarly, Katthagen et al. [69] showed superior performance with the use of femoral head allografts. Hsiao et al. [24] report significantly stiffer constructs with intramedullary cortical bone strut augmentation than the non-augmented constructs during cyclic compression tests.

Contrary results have also been reported. A recent study by Bulut et al. [47] performed abduction on three specimen groups: (1) control group with only locking plate implantation (2) locking plate implantation with fibular allograft augmentation along the shaft axis and (3) locking plate implantation with fibular allograft augmentation at 135° to support calcar and medial region. They reported no statistically significant difference in maximum loads and construct stiffness among the construct groups.

### Rigid vs. semi-rigid plates

Most of the complications associated with PHPs root back to the issue of poor implant anchorage, particularly in the elderly. A histomorphometric study by Hepp et al. demonstrated that current implants tend to target the central region of the humeral head where bone stock and bone quality are poor and the medial and dorsal aspects of the head should be targeted instead [64]. Maldonado et al. showed that in patients with osteoporosis, higher strain forces occur at the implant/bone interface compared to patients with healthy bone [49]. This may lead to early failure of relatively stiff constructs like angular stable plates or nails [49], especially in patients with reduced bone quality.

A number of patients admitted with proximal humerus fractures have good bone quality and with this patient population in mind, “rigid” implants were designed, that prevented micromotion of fracture to provide maximum construct stability. For the geriatric or the osteoporotic patient population, these rigid implants have higher risks of failure due to the poor bone-implant interface. Thus, a new series of implants, named “semi-rigid”, were designed. This design aims to increase the energy absorption by the implant and reduce the forces acting on the bone-implant interface by allowing some fracture motion.

The controversy arises on the matter of defining optimum stiffness of the implant. An excessively rigid implant poses a risk of developing extremely high peak stresses on the humerus which is not only mechanically but also biologically unsafe. On the other hand, implants with too low stiffness can lead to early failure and head migration due to poor mechanical support [97]. This dilemma is further complicated by the fact that the mechanical role of an implant changes during the fracture healing process and an intricate balance of implant elasticity is required for successful healing [98, 99].

Two of the semi-rigid implants for proximal humerus fractures are the Humerus Block (Synthes, Salzburg, Austria) and ButtonFix (Synthes, Solothurn, SO, Switzerland), intended to be minimally invasive fixations as they are accompanied by Kirchner wires and have dimensions smaller than conventional plates. Concerns have been raised regarding their elastic design which may be too elastic for the required healing and the potential risk of K-wires migration [100, 101].

As for their in vitro biomechanical performance, Duda et al. [65] reported higher compression, torsion and varus bending stiffness values for humeri treated with ButtonFix system as compared to those treated with Humerus Block system. Kralinger et al. [56] investigated the performance of locking compression plate with Humerus Block in cadaveric humeri under bending and torsion. While the semi-rigid Humerus Block would intuitively be less stiff (primary stability), they also performed cyclic tests to calculate the percentage reduction in load. Low reduction in load would suggest that the bone-plate construct is able to provide the stability needed for fracture healing (secondary stability). They reported that the locking plate construct was stiffer than the Humerus Block construct in all loading conditions but the load reduction was similar for both plates.

### Cement augmentation

Implant-related complications associated with PHPs, such as poor screw purchase, owe mostly to poor bone mineral density. One possible way to enhance implant anchorage in reduced bone stock is to increase the bone-implant interface by augmentation using bone cement. This method has already been established in other fractures of the human body including femur, tibial plateau and distal radius fractures [102–105].

Several in vitro biomechanical studies investigated the effect of implant augmentation on the management of proximal humerus fractures [39, 43, 44, 49, 58, 61, 87], all using either a calcium phosphate or a polymethyl methacrylate (PMMA) based cement. Gradl et al. [39] used self-setting calcium phosphate cement into all head screw holes of the AxSOS locking plate (Stryker, Kalamazoo, MI, USA). Significantly higher load to failure and stiffness were exhibited by cement-augmented specimens as compared to non-augmented specimens in cadaveric two-part fracture model. Similarly, Kwon et al. [61] demonstrated significantly higher failure torque and torsional stiffness as well as reduced interfragmentary motion for calcium phosphate cement-augmented cloverleaf (Synthes, Paoli, PA, USA) and blade (Synthes, Paoli, PA, USA)plate specimens, relative to the non-augmented specimens, using cadaveric three-part fracture model.

Kathrein et al. [87] augmented the four proximal screws of PHILOS plate with PMMA cement and demonstrated decreased per cycle motion and varus impaction of the humeral head during simulated 15°–45° cyclic abduction and adduction for 500 cycles in unstable two-part fractures. Roderer et al. [45] performed a mechanical assessment of the local bone quality in the screws’ directions before augmenting two anteriorly directed screws of PHILOS plate with PMMA bone cement to aim at the regions of lowest bone quality. Augmentation was found to significantly increase the number of load cycles to failure under varus bending, using a three-part fracture model.

Both Schliemann et al. [43] augmented the two anteriorly directed head screws with PMMA cement in DiPhis-H plate (Lima Corporate, San Daniele del Friuli, UD, Italy)while Unger et al. [58] augmented four screws with PMMA cement in the PHILOS plate. Schliemann et al. tested cadaveric humeri, treated for unstable three-part fractures, under varus bending and reported no significant increase in stiffness and failure loads but significant reduction in the bone-implant interface motion with augmentation. Unger et al. achieved a significantly higher number of load cycles until failure for the augmented group than the non-augmented group under cyclic varus bending and torsion in three-part fractures.

### Spatial subchondral support plate

As opposed to the PHILOS locking plate which is positioned higher on the greater tuberosity to form a neck angle that is almost at a right angle, the Spatial Subchondral support plate (S3 plate, Depuy, Warsaw, IN, USA) is placed 3 cm distal to the greater tuberosity to achieve a 135° neck angle. This placement aims to overcome the potential post-operative complication of subacromial impingement, one of the leading post-operative complications with PHILOS plate [106, 107]. Huff et al. [59] performed an in vitro biomechanical comparison of two-part fractures treated with an S3 plate and the Synthes locking plate and recorded higher stiffness for the S3 plate in torsion and varus and valgus bending tests but lower stiffness in extension and flexion bending, despite using a longer Synthes plate. Rose et al. [90], however, loaded constructs that had been treated for three-part fractures under simulated 10°–60° cyclic abduction and reported that the specimens stabilised with the S3 plate showed significantly higher displacement of greater tuberosity fragment and larger rotation of the head fragment than those repaired with conventional locking compression plate.

The S3 plate allows the use of smooth pegs rather than threaded screws for subchondral support, which has several theoretical advantages. Smooth pegs offer thicker core diameter for increased strength compared with screws and reduce the risk of articular penetration from humeral head collapse. A biomechanical study by Schumer et al. [51] was focused on this relationship and they detected no significant difference between smooth pegs and threaded screws in S3 plates for humeral head under cyclic compression and torsion in an unstable two-part fracture. Yamamoto et al. [60], however, recorded significantly less distal fragment displacement for cadaveric humeri treated with an S3 plate with smooth pegs than those treated with Synthes locking plate with threaded pegs. However, no significant difference was found for the two fixation methods in torsion tests. Because the authors used two different plates, it was difficult to determine whether this difference was due to the use of smooth pegs or the different screw orientation of the two plates or the more distal placement of the S3 plate.

While conventional locking plates have generally been reported to exhibit superior mechanical performances over non-locking plates, they have been shown to be statistically similar in performance to the S3 plate and plates with polyaxial locking screws. Cement augmentation and insertion of calcar support, both in form of screws or allografts, increase mechanical performance of plates whereas semi-rigid plates achieve reduced stiffness.

## Discussion

The discussion of the results has been divided based on the three research questions.

### Protocol design for in vitro biomechanical testing

There is a strong incentive for devising in vitro biomechanical studies that represent the in vivo situation more accurately, allowing one to foresee and prepare for the potential risk of failure. This has particularly been the case after the advent of locking PHPs. In vitro studies revealed superior biomechanical performance of locking PHPs over non-locking PHPs but clinical trials showed a different picture, laden with more cases of post-operative complications for locking plates. Interpreting results from clinical literature can be a challenge when assessing how well an in vitro biomechanical test represents clinical scenario. Multiple factors and uncertainties such as patient’s medical history and lifestyle contribute to final clinical outcome.

One approach to assess clinical applicability of an in vitro biomechanical test is to study the source of its loading conditions. Many in vitro studies took the step of applying physiological load values and angles that had been determined in previous studies. In particular, a large number of the studies involving combined bending and axial loading applied load 20° away from the shaft axis. This was because it had been found by Inman et al. [108] and Poppen and Walker [75] that loading at this angle produces the maximal axial and shear load to the humerus during a movement similar to the early active abduction. As for the glenohumeral contact forces, there is a notable example of their first in vivo measurements which were conducted by Bergmann et al. [76, 77]. Authors implanted telemeterised shoulder implants on a patient with arthrosis for the measurements of the post-implantation contact forces during activities of daily living and this formed the basis for six studies [39, 43–45, 68, 78]. Furthermore, there was a noticeable increase in the complexity and variety of in vivo-based loading conditions for the humerus-tendon testing studies despite the fact that there were only eleven of them. Prime examples of this were the studies by Voigt et al. where RASS (robot-assisted shoulder simulator) and hydraulic systems were used to control the pull of several muscles. Loading conditions for these muscles had been defined in previous studies such as those by Klages et al. [109] and Kedgley et al. [110]. Similar systems have also been used by Walsh et al. [81] and Osterhoff et al. [71]. These studies had the clear advantage of pulling the intact tendons often at the anatomical insertion points, as found in vivo.

The advantage of the humeral-tendon studies’ systems over the humerus-only studies’ loading is evident but many improvements are required to ensure that the systems are representing physiologically accurate conditions. For example, tests should first be made to fully understand the in vivo loading conditions of the humerus after fracture, not only before it. This includes taking into consideration the post-fracture scapulohumeral rhythm and its effects on the humerus. Contribution of individual muscles and the changes in magnitudes and directions of glenohumeral contact forces during everyday movements should also be considered.

Studies similar to that by Bergmann et al. [76], but on post proximal humerus fracture fixation scenario, will be highly valuable as they will provide us with the required loading conditions to aim for designing future test protocols. This could possibly be achieved by using implantable wireless (telemetry) motion and force sensing. In the literature, sensors have been implanted in shoulder arthroplasty systems to measures in vivo glenohumeral joint contact forces during activities of daily living [76, 79]. However, the implantation of these sensors in proximal humerus plates is yet to be reported in the literature. For proximal femoral fractures, however, there is a study which used a multi-channel telemetry system to record bending moments about the nail plate junction of implanted hip nails [111]. For proximal humerus fractures, such studies should be conducted for a larger number of patients than those reported and from various social and medical backgrounds. The number of movements performed should also be increased and varied according to patients’ lifestyles. This will provide us with data that can be used to simulate and verify different loading scenarios in vitro. It will also allow us to test humeri under pseudo-subject-specific loadings, based on humerus’ anthropometric data such as its geometry and dimensions, leading to loading conditions that are closer to cadaver donor’s own loading conditions. To the best of authors’ knowledge, studies basing the loading of humeri on their properties such as geometry and dimension are yet to be found, at least in the literature included in this review. This is largely because such properties have intentionally been kept constant by selecting similar humeri, to ensure a fair test.

With an appreciation of the uncertainties affecting clinical outcomes, many in vitro studies focused on complications that are most frequently reported in the clinical literature. For example, varus bending tests were very common among LT3 studies as it helps evaluate plates’ functionality for risk of varus collapse, a leading complication associated with PHPs. Katthagen et al. [68] conducted dedicated tests for screw perforation through the humeral articular surface. Such a problem-based approach ought to lead the design of new tests for other complications. It must be noted, however, that proximal humerus fractures are complex and these complications do not occur in isolation but are interlinked. For example, the poor implant-bone interface is commonly reported to be the reason behind most of PHPs’ post-operative complications. Thus, tests that not only assess the quality of this interface but also quantitatively define it, could potentially serve as a standard for predicting in vivo performance. We recommend testing PHPs for multiple, commonly reported complications.

PHPs aim to provide the balance in mechanical stimuli required for successful bone healing in the post-fracture scenario. To achieve this, their first role is to provide stability in the bone-plate construct, safeguarding it from abrupt and extreme changes to the local mechanical environment. The most common parameter to quantify this stability was the construct’s elastic stiffness which was calculated from the measured load and actuator displacement data. Advantages of stiffness as a stability parameter include its ease of calculation, requiring only the data collected from material testing machine, and its applicability to a wide range of axial, rotational and bending tests.

Given the prevalence of postoperative complications such as varus collapse, screw penetration and subacromial impingement, PHPs must maintain construct stability throughout patient’s life (Secondary stability). Conclusions derived solely from stiffness values calculated by loading in the elastic regime (primary stability) are therefore insufficient as they may not necessarily hold true over long-term, plastic loading. Instead, destructive tests to failure, either by static plastic loads or by a large number of cyclic loads, are needed. Common parameters calculated in the literature to assess constructs’ secondary stability include number of cycles to failure as well as load, displacement, stiffness and loss of stiffness both at failure and after a specified number of cycles. In the current review, for each of the six main controversial topics identified, there was at least one in vitro study that had tested the bone-plate construct to failure either by plastic or cyclic loading. In general, when assessing clinical applicability of in vitro studies, the results arising from such destructive tests should be prioritised over those from purely elastic tests.

The current decade has seen an increased use of 3D motion capture systems during in vitro testing. These can provide additional information regarding construct stability to that from stiffness alone. The interfragmentary displacements and rotations recorded from these systems can help locate regions of local instability within construct, especially in complex three-part fractures [57, 61, 82, 84]. Also, fracture gap distance and the relative displacements and rotations between bone and implant can been calculated from these systems [64, 69, 85–87]. These two parameters have the advantage that they are both already measured in clinic to assess post-operative stability of bone-plate constructs [112–114]. One example is the neck-shaft angle, which is the angle between the humerus’ anatomical neck and its shaft in the frontal plane commonly calculated from radiographs to assess construct’s varus stability. Several studies have stated that the normal anatomical neck-shaft angle is approximately 130°–135° [115, 116] whereas those less than or equal to 100° has been shown to predict failure [117]. A large number of in vitro studies based their failure criteria on the actuator load–displacement curves where their clinical interpretations were difficult to derive. We recommend the use of parameters such as neck-shaft angle, which have been directly derived from clinical assessments, as a basis for failure criteria.

It is known from in vitro tests on ovine tibia fractures that there exists an ideal window of stiffness within which successful fracture healing occurs [118]. While such a window for in vivo proximal humeral fractures is yet to be found, it will allow one to set a target range of construct stiffness values to aim for during in vitro tests. This debate of determining ideal stiffness for success fracture healing is the core issue in the controversy surrounding rigid and semi-rigid implants. Advocates of semi-rigid implants recommend a certain degree of plate flexibility, especially for osteoporotic bones, to allow sufficient fragment movements for bone healing [18, 56, 78]. Therefore, when testing semi-rigid plates, the emphasis is placed on the secondary stability of the constructs in order to determine if they can maintain their ability under plastic and cyclic loading [56]. It is also reported that rigid implants pose the risk of high stress concentration [119]. Thus, in addition to providing stability, PHPs are also required to distribute stresses and strains to the surrounding tissues as necessary for bone healing. While several mechano-regulation theories exist that relate mechanical stimulus to cell differentiation and tissue formation during fracture healing [99, 120–122], the current study revealed that the vast majority of in vitro studies focused only on the first role (stability) of PHPs. Strains were seldom measured, with Mathison et al. [33] being the noteworthy study using digital image correlation for measuring surface strains. Conducting in vitro tests with surface strain measurements can not only help identify regions of high stress concentrations but can also be used for validation of finite element models simulating them [123–125]. Since these models can be used to determine stress concentration inside the bone-plate construct, they can be used to develop better parameters to quantify the constructs’ mechanical performance. Strain measurements can also be used to identify regions of both plate and bone that are at a high risk of failure. This information is valuable for the development of plates that both stabilise the construct and better distribute the stress and strains across it.

For accurate recreation of the in vivo situation, it is crucial to use bio-realistic humerus specimens. A vast majority of studies used cadaveric specimens that theoretically have more accurate material properties than the synthetic ones which were used only in at least ten studies. However, in general, biological variability has been known to play a significant role in results and thus making it difficult to develop correlations and draw conclusions. Also, the chronological deterioration of the mechanical properties of cadavers is a well-documented phenomenon and need to be considered. Cartner et al. [126] investigated the effect of the post-freezing delay of fresh-frozen cadaveric femora on the pull-out strength of the implanted screw. Results showed that delaying the test for 50 h lead to a 9% drop in the pull-out strength relative to the control specimen which was tested after 16 h. Delaying the tests for 90 h resulted in approximately 30% decrease in the screw pull-out strength as compared to the control. It is therefore important to ensure that the results from the cadaveric specimens are not significantly influenced by this phenomenon. Furthermore, in order to characterise the typical elderly patient accurately, the use of osteoporotic surrogate bone specimens for biomechanical tests, should be considered.

Despite the popularity of cadaveric humeri, over 80% of all studies involved testing of humerus only, without any musculature or tendons attached, and performed one of the four loading types. The nature of these loading types was, to a large extent, mechanically very basic as they were based on simple axial loading, torsion and bending. Demand for more complex and physiologically accurate loading conditions dates back to pre-2000 studies [7, 25] and since then, several steps have been made. First of all, cyclic loading, which is more accurate to the in vivo conditions than static loading, was found to be a common mode of loading in literature. In case of Schumer et al. [51] and Dietz et al. [52], both the static and cyclic axial compression and torsion were imposed simultaneously. Simultaneous loading of the specimen with other loading types such as bending ought to be explored because real-life shoulder movements are a combination of these fundamental loading types. Furthermore, almost half of the studies involved multiple loading types with several using the same humeri specimen for all loading stages, again to make the tests as close to in vivo conditions as possible. It should be noted that despite conducting multiple types of loading, the studies were largely limited to the four basic loading types.

Vast majority of the studies involved testing of two or three plates. Tests for performance of multiple plates under same conditions, like Lever et al. [63], can be achieved by using a large sample size and by performing multiple pairwise comparisons. As far as the fracture models are concerned, most of in vitro studies cover the common two- and three-part fractures, with a few studies even introducing four-part fractures [72, 73]. However, the fracture patterns often varied and were according to different classification systems. There were only a few studies simulating more than one fracture patterns, notable examples of which are the works of Brunner et al. [78], Kathrein et al. [87] and Schliemann et al. [48]. Therefore, future biomechanical studies should include a variety of fracture patterns as well as plates.

### Controversial issues relating to proximal humerus plates

Many questions remain unanswered with regards to technologies and techniques relating to PHPs. For example, results from the biomechanical studies tend to favour the insertion of calcar region support.

This can be in the form of inferomedial screws, which has been shown in several studies [37, 38, 41, 70] to significantly improve construct mechanical performance. However, a detailed investigation of the importance of support to the calcar region in comparison with other cephalic regions for construct stability is required. This will also highlight the mechanical importance of different areas and screws of the plates and thus could guide the design process of novel PHPs. Such an investigation, particularly if conducted for multiple complex fracture types is also of high clinical value as it could support clinicians in making pre-operative decisions. Mechanical advantages of employing allografts in open reduction internal fixation are supported in several studies [27, 33, 34, 69, 71], under a variety of loading conditions [27, 33, 34, 69, 71]. However, these studies on the applications of allografts are limited to two-part fracture patterns. Thus, their effects and implications under complex fractures such as three-part fractures also ought to be explored. Furthermore, contradicting results have been reported by Bulut et al. [47], which require further investigation.

A total of seven studies [25, 31, 46, 55, 61–63] have tested the blade plates but they have all been limited to the traditional non-locking blade plates. With the recent advent of new hybrid locking blade plates such as the Equinoxe Fx plate (Exactech, Gainesville, FL, USA) that are locking and include the option of blade insertion, a biomechanical comparison of their performance against other leading plates was not found in the literature.

In response to the clinical problems reported for locking plates, several plates have been designed, based on new technologies. One such plate is the NCB plate (Zimmer, Warsaw, IN, USA) which relies on polyaxial screw systems for support. Biomechanical studies, however, show varying results. For example, Zettl et al. [28] demonstrated that NCB plate can achieve statistically similar performance to a PHILOS plate (monoaxial screw system) by using fewer but thicker screws for NCB plate. Erhardt et al. [38] report similar performance with the insertion of polyaxial screws instead of monoaxial ones. Further investigation is also required to determine their efficacy in comparison with the traditional monoaxial screw systems. In a similar spirit, the S3 plate was designed with complications such as subacromial impingement and screw perforation in mind. So, the plate was designed to be placed more distally than most other locking plates like PHILOS plate but also with the option of using smooth pegs. Yamamoto et al. achieved superior biomechanical characteristics superior with the use of S3 plate with smooth pegs than a PHILOS plate with threaded screws. On the other hand, Schumer et al. [51] reported similar results with smooth pegs and threaded screws on S3 plate. Therefore, further studies are required since the in vitro results on this issue remain insufficient to delineate the superior design.

## Conclusions

Most studies performed mechanically simple loadings based on axial loading, torsion and bending and tested two and three-part fractures. Elastic stiffness served as a good general-purpose performance parameter for quantifying early construct stability while strength and fatigue parameters better represented long-term functional stability. Stress and strain distributions directly influence fracture healing process but were seldom measured. Locking plates were generally mechanically superior to non-locking ones but conflicting results were reported when compared to non-locking blade plates. Mechanical benefits of polyaxial locking screws over monoaxial ones remain unclear. Medial support insertion, both in form of screws and allografts, and cement augmentation generally improved mechanical stability, with a few studies reporting no significant improvement. Semi-rigid implants, Humerus Block and ButtonFix, exhibited lower torsional and bending stiffness than locking plates. Comparisons between S3 plate and conventional locking plates and that between threaded screws and smooth pegs reported conflicting results. It is hoped that the review will aid development of future in vitro and in silico biomechanical studies. We recommend studying this review alongside clinical reviews when evaluating plates’ performance and in vitro tests’ clinical applicability. This will guide the design of better plates and studies with more accurate loading conditions and parameters and lead to their standardisation.

## Additional file

**Additional file 1.** Details of the implants, bone specimen, loading conditions and the measurements undertaken in the biomechanical studies.
