# Supplementary material for: Biomechanical analysis of plate systems for proximal humerus fractures: a systematic literature review
Source: Biomed Eng Online. 2018 Apr 27;17:47. doi: 10.1186/s12938-018-0479-3 (PMC5923007; doi:10.1186/s12938-018-0479-3)
Supplement: Supplementary file 1 — Additional file 1. Details of the implants, bone specimen, loading conditions and the measurements undertaken in the biomechanical studies. [file 12938_2018_479_MOESM1_ESM.doc]

| **Study** | **Implant** | **Bone Specimen** | **Loading Conditions** | **Measurements** |
| --- | --- | --- | --- | --- |
| **TYPE 1 LOADING ONLY** | | | | |
| Seide et al., 2007 | TIFIX internal fixator plate (LITOS) | Human cadaver humeri | **Fracture:** Two-part fracture, 10 mm subcapital osteotomy  **Static Test:** Axial compression at 5 mm/min until failure  **Dynamic Test:** Cyclic axial compression with 10 N preload and 80 N maximal load at 5 Hz until 1,000,000 cycles  **Failure:** For static test, clear deviation from linearity on load-displacement curve; for dynamic test, automatic stopping of testing machine due to control instability | **Static Test:** Recorded force, displacement, determined linear elastic stiffness and load at failure  **Dynamic Test:** Recorded force-displacement data, determined number of cycles to failure and maximum plastic deformation |
| Zettl et al., 2011 | PHILOS plate (Synthes)  Non-contact bridging plate (Zimmer) | Human cadaver humeri | **Fracture:** Two-part AO-A3 fracture, 10 mm medial wedge osteotomy  **Cyclic Test:** 90, 180 and 450 N cyclic axial compression at 1 Hz for 200 cycles, then 0-450 N at 1 Hz for 2000 cycles  **Static Test:** Axial compression at 1 N/s until failure  **Failure:** >30% pressure drop, 30 mm deformation or observation of screw cut-out | **Cyclic Test:** Recorded force, displacement, determined maximum plastic deformation  **Static Test:** Recorded force, displacement, determined force at failure |
| Bae et al., 2011 | PHILOS plate (Synthes) | Human cadaver humeri | **Fracture:** First osteotomy in line with humeral anatomical neck and second 1 cm distal to the inferomedial aspect of articular cartilage of humeral head  **Dynamic Test:** 10-80 N cyclic axial compression at 5 Hz for 1,000,000 cycles  **Static Test:** Axial compression at 5 mm/min until failure  **Failure:** Clear deviation from linearity on load-displacement curve | **Dynamic Test:** Recorded force and displacement, determined construct displacement after 1,000,000 cycles (difference in actuator position before and after cyclical loading)  **Static Test:** Recorded force and displacement**,** determined linear elastic stiffness and failure load |
| Wallace et al., 2012 | Proximal humerus locking plate (Synthes) | Human cadaver humeri | **Fracture:** Two-part comminuted fracture, standard 15 mm resection osteotomy at humeral surgical neck  **Static Test:** Axial compression at 5 mm/min until failure  **Failure:** Screw pull-out or complete closure of fracture gap | Recorded force and displacement, determined load at failure, stiffness, maximum load, cortical thickness |
| Gradl et al., 2012 | Humerus Tele Screw plate (M.O.R.E. Medical Solutions)  PHILOS plate (Clinical House) | Human cadaver humeri | **Fracture:** Three-part (Neer IV/3) fracture, 5 mm osteotomy gap simulating metaphyseal comminution  **Cyclic Test:** 25-50 N sinusoidal axial compression at 1 Hz for 50 cycles, then increased maximum load by 50 N up to 3000 N every 50 cycles  **Failure**: 20 mm humeral head displacement | Recorded force, displacement and number of cycles, determined stiffness and load at failure |
| Instrum et al., 1998 | Semitubular blade plate  AO T-plate (Synthes) | Human cadaver humeri | **Fracture:** Two-part fracture, transverse osteotomy created 10 mm beneath the inferior screw of the head fragment  **Static Test 1:** Axial tension at 20 mm/min until failure  **Cyclic Test:** 90-180 N sinusoidal axial tension at 1/6 Hz for 1500 cycles  **Static Test 2:** Axial tension at 20 mm/min to failure  **Failure**: N/A | Recorded force and displacement, determined yield load and load to failure |
| Hsiao et al., 2017 | Custom-made locking plate | Human cadaver humeri | **Fracture:** Two-part fracture, 10-mm gap osteotomy created at surgical neck level  **Cyclic Test:** Cyclic axial load applied to 450 N at 1 Hz for 6000 cycles with 10 N pre-load  **Static Test:** Axial tension at 5 mm/min to failure  **Failure**: (During cyclic tests) sudden drop in load-displacement curve  (During static tests) maximum applied force or closure of osteotomy gap | **Cyclic Test:** Recorded load and displacement data for each cycle  Determined peak-to-peak (inter- cyclic) displacement and cumulated deformation at specific cycles (100th and every 1000 cycles)  **Static Test:** Determined failure load |
| **TYPE 2 LOADING ONLY** | | | | |
| Weinstein et al., 2006 | Proximal humerus locking compression plate (Synthes)  Angle blade plate (Synthes) | Human cadaver humeri | **Fracture:** Three-part fracture, first osteotomy across surgical neck and second osteotomy, from lateral aspect of bicipital groove to inferior to tuberosity flare (separating greater tuberosity from humeral head)  **Static Test:** 0-5 Nm axial external rotation torque at 0.5°/s  **Cyclic Test:** 0-5 Nm axial cyclic external rotation torque at 0.5 Hz until failure  **Failure:** 30^o^ humeral head rotation with respect to shaft or 10,000 load cycles | **Static Test:** Recorded torque, rotational displacement, determined torsional stiffness  **Cyclic Test:** Recorded number of cycles and rotation data, determined maximum rotational deformation |
| Foruria et al., 2010 | PHILOS plate (Synthes)  Proximal humerus nail with spiral blade (Synthes) | Human cadaver humeri | **Fracture:** Two-part fracture, 0.5 cm osteotomy, 1 cm distal to inferomedial aspect of head articular cartilage and perpendicular to shaft  **Cyclic Test:** Sinusoidal cyclic rotational torque between 0.25 Nm internal rotation and 1112 Nm external rotation at 1 Hz for 10,000 cycles  **Static Test:** External rotation at 30 o/min loading rate until failure  **Failure:** For cyclic test, implant and bone dissociation, appearance of new fracture line, >45^o^ angular displacement  For static test, dissociation between implant and bone, appearance of new fracture line, or implant fracture | **Cyclic Test:** Recorded torque, interfragmentary angular displacement, determined maximum angular displacement between proximal and distal fragments  **Static Test:** Recorded torque and interfragmentary angular displacement, determined torsional stiffness, torque at failure and energy absorbed until failure (area under torque vs. displacement plot) |
| **TYPE 3 LOADING ONLY** | | | | |
| Mathison et al., 2010 | Proximal humerus locking plate (Synthes) | Human cadaver humeri | **Fracture:** Two-part fracture, 10 mm wedge-shaped osteotomy at level of surgical neck    **Static Test:** Shaft displaced in medial direction (varus bending) at 4 mm/min until failure  **Failure**: Humeral head breaking apart or shaft snapping | Recorded force, relative movement between the fracture surfaces (using digital image correlation), determined stiffness and failure load |
| Chow et al., 2012 | Locking compression plate (Synthes) | Human cadaver humeri | **Fracture:** Two-part fracture, 1 cm wedge-shaped osteotomy at the level of surgical neck, proximal osteotomy cut made transversely at level of inferomedial margin of the articular surface  **Cyclic Test:** 0-110 N varus load at a rate sufficient to create 600 mm/min until failure  **Failure:** Varus collapse or 25000 load cycles | Recorded number of cycles, initial load position, position at peak load application (110 N) for every cycle and displacement at peak load  Determined average deformation per cycle, number of cycles needed to cause 1 mm deformation, number of cycles to failure |
| Weeks et al., 2013 | Locking compression plate (Synthes) | Same as above (Chow et al., 2012) | Same as above (Chow et al., 2012) | Same as above (Chow et al., 2012) |
| **TYPE 4 LOADING ONLY** | | | | |
| Koval et al., 1996 | AO T-plate (Synthes)  Tension banding fixations  Kirschner wires fixations  AO Schanz pins fixations  Ender intramedullary nails (Richards) | Human cadaver humeri | **Fracture:** Two-part fracture, osteotomy created at greater tuberosity base at 10^o^ oblique angle in medial-inferior direction  **Static Test:** Humeral shaft oriented at 20^o^ abduction and vertically displaced at superior aspect of humeral head 1 cm medial from its lateral edge at 10 cm/min until failure  **Failure:** Marked decrease or discontinuity in force-displacement curve | Recorded force and displacement, determined ultimate load, stiffness |
| Chudik et al., 2003 | ASIF T-plate (Synthes)  Experimental plate | Human cadaver humeri | **Fracture:** Two-part fracture, first osteotomy non-comminuted 10° oblique at surgical neck fracture (type 11-A3) and second, a 10° at oblique at surgical neck fracture with removal of a 1 cm medially based wedge of cortex  **Axial Preloading (Humeri-only):** Axial compression loading up to 750 N  **Static Test (Humeri with implant):** Humeral shaft oriented at 20^o^ abduction, loaded at 10 cm/min until failure  **Failure**: marked decrease/discontinuity in load-displacement curve or >1 cm displacement | **Axial Preloading:** Recorded force and displacement, determined stiffness  **Static Test:** Recorded force and displacement, determined stiffness, stiffness ratio (static test stiffness/preloading stiffness), displacement after 0.3 and 0.6 kN (physiological loads) and displacement, ultimate load and energy at failure |
| Ponce et al., 2013 | PHILOS plate (Synthes) | Human cadaver humeri | **Fracture:** Three-part fracture involving the surgical neck and greater tuberosity  **Static Test:** Constructs fixed at 20^o^ from vertical, vertical load at superior aspect of humeral head at 10 cm/min until failure  **Failure:** For comminuted specimen, medial cortical defect closure | Recorded force and displacement, determined stiffness and displacement, load and energy to failure |
| Gradl et al., 2013 | AxSOS locking plate (Stryker) | Human cadaver humeri | **Fracture:** Two-part fracture featuring metaphyseal comminution, 5 mm wedge osteotomy at surgical neck level  **Cyclic Test 1:** Construct fixed at 20^o^ of lateral angulation. 50-100 N sinusoidal vertical compressive load at 0.2 Hz for 50 cycles  **Cyclic Test 2:** Construct fixed at 20^o^ of lateral angulation and sinusoidal vertical compressive displacement (6 mm/min rate, 0.2 mm maximum deflection, 0.2 Hz frequency) applied until failure. Preload kept fixed at 50 N and peak load increased 100 N every 50 cycles until failure or until 5000 N load.  **Failure:** Complete osteotomy gap closure, 15^o^ angular displacement in unloading condition, sudden decrease in recorded force | Recorded force and displacement, determined axial stiffness and load to failure |
| Roderer et al., 2013 | PHILOS plate (Synthes) | Human cadaver humeri | **Fracture:** Three-part fracture, 10 mm horizontal gap below anatomical neck and greater tuberosity osteotomy  **Cyclic Test:** Cyclic loading at 0.25 Hz at 15-50 N with upper load increasing at 0.035 N/cycle until failure  **Failure:** >0.5^o^ increase of varus angular tilting within 100 load cycles at lower magnitude (constant 15 N) | Recorded plate and humeral head relative motion using ultrasound-based 3D motion analysis system, determined number of cycles to failure |
| Roderer et al., 2013 | PHILOS plate (Synthes) | Human cadaver humeri | **Fracture:** Two-part fracture (OTA type 11-A2), osteotomy with medially ascending 10 mm fracture gap  **Static Test:** Construct fixed at 25^o^ of lateral angulation. Axial compression loading increased at 0.02 mm/s until 200 N  **Cyclic Test:** Construct fixed at 25^o^ of lateral angulation. Cyclic compression loading at 1 Hz with preload fixed at 50 N and peak load increasing at 0.05 N/cycle. Loaded until failure or >15 mm actuator displacement  **Failure:** 2 mm head migration | **Static Test:** Recorded axial force and axial displacement, determined axial stiffness  **Cyclic Test:** Determined number of cycles to failure and humeral head mi­gration (using fluoroscopic assessment) after set 1000, 2000 and 3000 cycles |
| Yoon et al., 2014 | 3.5 mm Locked fixed angle plate  4.5 mm Locked fixed angle plate  Intramedullary Nail with locked screw  Intramedullary Nails with fixed angle blade | Human cadaver humeri | **Fracture:** Two-part fracture (AO/OTA 11-A3), first osteotomy at surgical neck at a 10^o^ oblique angle directed in a medial-inferior direction and second a 1 cm medially-based wedge of cortex at fracture site  **Static Test:** Construct fixed at 20^o^ of abduction and vertical compressive displacement applied at 10 cm/min until failure  **Failure**: marked decrease/discontinuity in load-displacement curve | Recorded force and displacement, determined stiffness and ultimate load to failure |
| Gillespie et al., 2009 | Locking compression plate (Synthes)  90° blade plate (Zimmer)  Periarticular standard proximal humerus plate (Zimmer) | Human cadaver humeri | **Fracture:** Three-part fracture (AO 11.B2), osteotomies of surgical neck (10°, oblique) and greater tuberosity (5 mm wide)  **Cyclic Test 1:** Construct fixed at 20^o^ of abduction and 10-110 N vertical compression applied at 1 Hz for 200 cycles  **Cyclic Test 2:** Construct fixed at 20^o^ of abduction and 10-410 N vertical compression at 1 Hz for 50 cycles or until failure  **Failure:** Fixation loss between some aspect of implant and proximal humerus | Recorded force and vertical displacement (using extensometer), determined linear elastic stiffness for each cycle |
| Hymes et al., 2013 | PERI-LOC plate (Smith & Nephew) | Human cadaver humeri | **Fracture:** Two-part fracture (OTA 11-A3) created by removing a 1 cm segment of bone below the surgical neck  **Cyclic Test:** 500±100 N cyclic compressive loads applied 30^o^ posteromedial to the anteroposterior (in plane of rotator cuff pull) at 2 Hz for 15,000 cycles (or until failure)  **Failure:** 20 mm or rapid (due to sudden bone fracture) actuator displacement | Recorded displacement, number of cycles, number of microcracks (using acoustic emission), determined number of cycles to failure |
| Erhardt et al., 2012 | Non-contact bridging plate (Zimmer) | Human cadaver humeri | **Fracture:** Two-part fracture (OTA/AO type C) at anatomic neck, second parallel cut 8 mm proximal to first  **Cyclic Test:** Construct fixed at 30^o^ flexion and 30^o^ abduction and 5-200 N compressive load for 200 cycles with load increasing 100 N every 200 cycles until failure  **Failure:** Visible screw perforation of at least one screw | Recorded force and displacement, determined linear elastic stiffness, number of cycles to failure and load to failure |
| Schliemann et al., 2015 | CFR-PEEK DiPhos-H plate (Lima Corporate) | Human cadaver humeri | **Fracture:** Three-part fracture, 10 mm gap osteotomy 10 mm below anatomical neck and second at greater tuberosity  **Cyclic Test:** Construct fixed at 25^o^ lateral angulation and vertical compressive load applied at 1 Hz for 10,000 cycles with load increase of 0.035 N/cycle up to 400 N maximum load after 10,000 cycles  **Static Test:** Construct fixed at 25^o^ lateral angulation and vertical compressive displacement applied at 5 mm/min until failure  **Failure**: Fracture gap closure, plate breakage, head fragment dislocation (assessed macroscopically) or >0.5^o^ increase in angular tilting within 100 load cycles at 15 N lower load | Recorded relative motion between lesser tuberosity and shaft and proximal and distal plate aspects (3D motion capture system), determined stiffness and load to failure |
| Burke et al., 2014 | PHILOS plate (Synthes) | Synthetic humeri (Sawbones 4^th^ generation, composite) | **Fracture:** Three-part fracture  **Cyclic Test:** 532.6 N compressive load applied at 1 Hz for 1000 cycles and then increased until failure  **Failure**: N/A | Recorded movement of greater tuberosity, articular surface and humeral diaphysis relative to the humeral shaft at 250, 500, 750, and 1000 cycles (using optoelectronic camera system), determined load to failure |
| Bulut et al., 2017 | Miss LC proximal humerus plate (TST, Istanbul, Turkey) | Synthetic humeri (Sawbones model 1028-64, polyuerathane foam) | **Fracture:** Two-part proximal neck fracture  **Static Test:** Humeral shaft oriented at 20^o^ abduction and vertically displaced at superior aspect of humeral head 1 cm medial from its lateral edge at 10 cm/min until failure  **Failure:** N/A | Measured displacements between fracture ends with a camera and extensometers and calculated the gauge length elongation. Also recorded maximum loads and maximum displacements. |
| Schliemann et al., 2017 | CFR-PEEK DiPhos-H plate (Lima Corporate)  PHILOS (Synthes) | Human cadaver humeri | **Fracture:** Two-part fracture model simulated by performing a 10-mm gap osteotomy 10 mm below anatomical neck. Three-part fracture model simulated by performing additional osteotomy at greater tuberosity  **Cyclic Test:** Humeri oriented at 25^o^ lateral angulation. Cyclic sinusoidal compression loading applied at 1 Hz for 10,000 cycles or until construct failure. Base load constant at 15 N and peak load increased continuously from 75 N at 0.035 N/cycle. After 10000 cycles, peak load was 400 N.  **Failure: >**0.5­^o^ increase in angular tilting more than within 100 load cycles at 15 N | Actuator stopped after 500 cycles, at 15 N, to allow photo documentation  Recorded relative motion between lesser tuberosity and shaft and proximal and distal plate aspects (3D motion capture system)  Determined plate bending (angular change between the two plate markers)  Determined stiffness and load to failure |
| **TYPE 1 & 2 LOADING** | | | | |
| Hessmann et al., 2005 | PHILOS 130^o^ plate (Stratec)  PHILOS 95^o^ plate (Stratec)  AO T-plate (Synthes)  Intramedullary nail with locking spiral blade (Synthes) | Human cadaver humeri | **Fracture:** Two-part fracture (AO 11-A3), wedge osteotomy with 8 mm medial defect at level of the surgical neck and transverse osteotomy started medially, inferior to articular cartilage  **Cyclic Test 1:** 10-120 N sinusoidal axial compression at 0.1 Hz for four cycles, then torsion at 0.4-2.5 Nm for four cycles  **Cyclic Test 2:** 200 cycles of sinusoidal axial compression and torsion under same loading conditions as cyclic test 1  **Static Test:** Axial load for 45s to 500 N until failure  **Failure:** Irreversible osteotomy gap closure in unloaded condition, 15^o^ irreversible angular displacement or sudden deviation from linearity on load-displacement curve | Recorded force, torque and displacement, determined stiffness, load to failure, plastic deformation assessed under preload conditions after 20, 100, and 200 cycles |
| Schumer et al., 2010 | S3 plate (DePuy) | Human cadaver humeri | **Fracture:** Two-part fracture (OTA type 11-A3.3), 1 cm surgical neck gap osteotomy  **Cyclic Test 1:** ±2 Nm cyclic torsion at shaft distal end at 1 Hz with 30 - 50 N simultaneous compressive axial load for 3000 cycles  **Cyclic Test 2:** Cyclic test 1 repeated for torsion at ±5 Nm for additional 3000 cycles  **Static Test:** Axial compression in axial compression at 20 N/s until failure  **Failure:** Major drop in applied load or closed head and shaft gap with plate plastic deformation | **Cyclic Test 1:** Recorded angular rotation, axial displacement, load and torque every 100 cycles, determined mean rotational displacement for each 3000-cycle testing period  **Cyclic Test 2:** Determined number of cycles to failure for specimens failing before completing 3000 cycles  **Static Test:** Determined load at failure |
| Dietz et al., 2012 | PHILOS plate (Synthes)  Retron nail (Tantum) | Human cadaver humeri | **Fracture:** Two-part fracture (AO 11-A3), wedge osteotomy with 10 mm defect at humeral neck and base of resected triangle at medial side  **Cyclic Test 1:** 10 N axial compressive loading applied on humeri only (with no implantation) for 15s with 0.5-2 Nm sinusoidal torsion torque applied simultaneously for 8 cycles, then, torque set constant at 0.5 Nm with 120 N sinusoidal axial compressive load applied simultaneously for 8 cycles  **Cyclic Test 2:** Same loading conditions as cyclic test 1 applied on implanted humeri for 4 cycles  **Cyclic Test 3:** Same loading conditions as cyclic test 1 applied on implanted humeri for 1000 cycles  **Failure:** Irreversible osteotomy gap closure, peri-implant fracture, sudden deviation from linearity on load displacement curve | **Cyclic Test 1:** Recorded force and displacement, determined stiffness (axial and torsional stiffness)  **Cyclic Test 2 & 3:** Determined the axial and torsional stiffness and percentage stiffness loss (compared to initial stiffness) |
| Maldonado et al., 2003 | Locking compression plate (Mathys) | Human cadaver humeri | **Fracture:** Two-part fracture, 5 mm osteotomy at level of surgical neck  **Static Test:** 0.5 mm axial compressive displacement at 5 mm/min and 4^o^ torsional torque with 25 N axial compressive preload at 50 o/min  **Failure:** N/A | Recorded fragment relative movements (using optical measurement system), determined axial and torsional stiffness |
| **TYPE 2 & 3 LOADING** | | | | |
| Fuchtmeier et al., 2007 | Sirus nail (Zimmer)  Proximal humerus nail with spiral blade  AO T-plate (Synthes)  PHILOS plate (Synthes) | Human cadaver humeri | **Fracture:** Two-part (AO 11 A3), osteotomy below humeral head at level of surgical neck to create a 5 mm defect  **Cyclic Bending Test:** Load of zero to a limit load (load required to create 7.5 Nm bending moment at osteotomy) applied at 1 mm/min for 5 cycles  **Cyclic Torsional Test:** 0-100 N (8.3 Nm moment at osteotomy) applied at 1 mm/min for 5 cycles  **Failure:** N/A | Recorded force and displacement between osteotomy centre and distal point of load transmission, determined deformation and angular deflection after a set number of cycles, bending and torsional stiffness determined and loss of bending and torsional stiffness |
| Yamamoto et al., 2013 | Locking compression plate (Synthes)  S3 (Depuy) | Human cadaver humeri | **Fracture:** Two-part fracture, 10 mm wedge surgical neck gap osteotomy  **Cyclic Bending Test:** Cyclic distal shaft load with cantilever at 5 mm/s for 10,000 cycles, producing 0-7.5 Nm bending moment  **Cyclic Torsion Test:** ±2 Nm cyclic distal shaft torsion at 20 o/s for 10,000 cycles  **Failure:** N/A | Recorded distal fragment vertical displacement and angular rotations and number of cycles, determined maximum displacement |
| Edwards et al., 2006 | Proximal humerus nail (Synthes)  Locking compression plate (Synthes) | Human cadaver humeri | **Fracture:** Two-part fracture of surgical neck created at greater tuberosity base at 10° oblique angle, directed in a medial-inferior direction with excision of 10 mm bone wedge to simulate comminution  **Cyclic Bending Test:** Cyclic cantilever load to produce 0-7.5 Nm varus bending moment at fracture site for 5000 cycles  **Cyclic Torsion Test:** ±2 Nm of cyclic torsional torque to distal fragment for 5000 cycles  **Bending Test 2:** 125 mm bending displacement  **Torsion Test 2:** Torsional load until failure  **Failure:** N/A | **Cyclic Bending Test:** Recorded force and displacement, determined bending stiffness, mean and maximum displacement for each 1000-cycle interval and number of cycles and the maximum displacement until failure  **Cyclic Torsion Test:** Recorded torque and angular rotation, determined torsional stiffness, mean and maximum angular rotation for each 1000-cycle interval and number of cycles and the maximum angular rotation until failure  **Bending Test 2:** Determined displacement and torque at maximum displacement  **Torsion Test:** Determined torque and angle at failure |
| Siffri et al., 2006 | Locking compression plate (Synthes)  Fixed angle blade plate (Synthes) | Human cadaver humeri  and synthetic humeri (Sawbones 2^nd^ generation) | **Fracture:** Two-part fracture (OTA type 11 A3), transverse osteotomy at surgical neck  **Bending Test:** Cyclic distal shaft load to produce 0-7.5 Nm bending moment for 10,000 cycles  **Torsion Test:** Cyclic shaft load to produce ±2 Nm torsional torque for 5000 cycles  **Failure:** N/A | **Bending Test:** Recorded force, displacement and number of cycles  **Torsion Test:** Recorded number of cycles and angular rotation of distal fragment with respect to fixed humeral head |
| Huff et al., 2013 | Locking compression plate (Synthes)  S3 plate (DePuy) | Human cadaver humeri  and synthetic humeri (Sawbones model 1028 polyuerathane foam) | **Fracture:** Two-part fracture, transverse cut at surgical neck 5 cm distal from highest point of humeral head with excision of 10 mm bone block  **Cyclic Bending Test:** ±5 mm distal shaft displacement at 1 mm/s for 100 cycles, in sagittal plane for flexion/extension bending and frontal plane for varus/valgus bending  **Static Bending Test:** Varus loading at 1 mm/s until failure  **Cyclic Torsion Test:** ±8^o^ shaft rotation at 1 o/s for 100 cycles  **Static Torsion Test:** External loading (positive direction) at 1 o/s until failure  **Failure:** N/A | **Cyclic Bending Test:** Recorded force and displacement to determine varus, valgus, flexion and extension peak load at first and last cycle  **Static Bending Test**: Determined varus bending stiffness  **Cyclic Torsion Test:** Recorded angular position and torque, determined peak torques of first and last cycles in internal and external rotation  **Static Torsion Test**: Determined torsional stiffness |
| Unger et al., 2012 | PHILOS plate (Synthes) | Human cadaver humeri | **Fracture:** Three-part fracture, 10 mm horizontal gap osteotomy beneath anatomical neck and greater tubercle osteotomy  **Cyclic Bending Test:** 15-50 N sinusoidal varus load at 0.25 Hz with upper load increasing by 0.035 N/cycle until screw bone fixation failure  **Cyclic Torsion Test:** ±0.5 Nm sinusoidal torsional torque at 0.25 Hz upper load increasing by 0.035 N/cycle in both directions until screw bone fixation failure, with 20 N constant axial compressive load  **Failure:** For bending test, >0.5^o^ increase of varus angular tilting within 100 load cycles at the lower load magnitude (constant 15 N)  For torsion tests, >4^o^ axial rotation during one load cycle | **Cyclic Bending Test:** Recorded relative motion between plate and humeral head using 3D motion analysis system, determined elastic and plastic deformation in varus angular tilting  **Cyclic Torsion Test:** Determined maximum humeral head rotation for a set interval of cycles and number of cycles and torque to failure |
| Ruch et al., 2000 | Tension band wire fixation with Enders intramedullary nail  Modified cloverleaf plate  Intramedullary device with proximal interlocking screws (Polarus) | Human cadaver humeri | **Fracture:** Three-part fracture, involving humeral neck and greater tuberosity with 2 mm metaphyseal bone resected during osteotomy to simulate bone loss  **Static Bending Test:** Flexion, extension, varus, and valgus loading up to 5 mm at 1 mm/s with 200g preload and each specimen deflected twice in each direction  **Static Torsion Test:** Internal shaft rotation at 0.5 o/s until 5 Nm torque or 30^o^ of rotation, repeated for opposite direction  **Failure:** N/A | **Static Bending Test:** Recorded force and displacement, determined bending stiffness  **Static Torsion Test:** Recorded torque and rotation, determined torsional stiffness |
| Kitson et al., 2007 | Intramedullary nail (Austofix)  Humeral locking plate (Mathys Medical) | Human cadaver humeri | **Fracture:** Three-part fracture, creating greater and lesser tuberosity and head and shaft fragments  **Static Bending Test:** Valgus, varus, extension and flexion loading at 1 mm/s with 2 N (200 g force) preload up to 5 mm  **Static Torsion Test:** Torsion at 0.5°/s with 0.2 Nm preload up to 6.5 Nm (within elastic region)  **Static Valgus Bending to Failure Test:** Valgus load until failure  **Failure:** N/A | **Static Bending Test:** Determined stiffness  **Static Torsion Test:** Recorded torque and rotation, determined torsional stiffness  **Static Valgus Bending to Failure Test:** Determined load at failure |
| Roderer et al., 2011 | Non-contact bridging plate (Zimmer) | Human cadaver humeri | **Fracture:** Three-part fracture (AO/ASIF 11-B1), first, osteotomy at the surgical neck and second from lateral aspect of bicipital groove to just inferior to the flare of the tuberosity, separating greater tuberosity from humeral head  **Cyclic Test:** Pure moments around three axes (x = varus/valgus, y = flexion/extension, z = torsion) at 2, 3.5, 5 and 7.5 Nm at 0.5°/s for 100 cycles  **Failure:** >30^o^ angular displacement | Recorded interfragmentary motion between humeral head and shaft after 0, 10, 20, 30, 50, 70 and 100 cycles, determined number of load cycles until failure |
| Kralinger et al., 2008 | Humerus locking compression plate (Synthes)  Humerus Block (Synthes)  Intramedullary claw (ITS) | Human cadaver humeri | **Fracture:** 5 mm slice resected at surgical neck  **Static Varus Bending Test:** Cantilever bending with torque of 1.5 Nm to PHP and HB and 0.75 Nm to IMC (simulating supraspinatus pull)  **Static Torsion Test:** Torque along humeral shaft axis of 1.5, 0.7 and 0.45 Nm for PHP, HB and IMC respectively  **Static Medial Shearing Test:** Muscle pull of pectoralis major simulated, distal fragment fixed and 30 N applied against head fragment to simulate pectoralis major pull  **Cyclic Test:** Varus bending (similar to static varus bending test) at 0.5 Hz for 100 cycles  **Failure:** N/A | Recorded relative movement of fracture segments using ultrasound based 3D motion analysis system  **Static Varus Bending Test:** Determined bending stiffness  **Static Torsion Test:** Determined torsional stiffness  **Static Medial Shearing Test:** Determined stiffness  **Cyclic Test:** Determined minimum and maximum force and percentage load reduction per 1000 cycles |
| **TYPE 2 & 4 LOADING** | | | | |
| Carrera et al., 2008 | Conventional 90^o^ angular blade plate  Modified 90^o^ angular blade plate | Synthetic humeri (wood) | **Fracture:** Two-part fracture, oblique 110^o^ to the diaphysis longitudinal axis  **Static Test 1:** Humeral shaft oriented at 20^o^. Flexion load at 5 mm/min until maximum load tolerated by plates  **Static Test 2:** Torsional load at 5 mm/min until maximum torque tolerated by plates  **Failure:** Drop or marked interruption of load associated with an abrupt slide of proximal segment of wooden model from its initial position | **Static Test 1**: Recorded force and displacement, determined bending strength (maximum load) and stiffness  **Static Test 2:** Determined torsional stiffness, maximum torsion and angular dislocation at the point of maximum torsion |
| Kwon et al., 2002 | Cloverleaf plate (Synthes)  Angled blade plate (Synthes)  Kirschner wires with cement (Synthes) | Human cadaver humeri | **Fracture:** Three-part fracture, surgical neck and greater tuberosity osteotomy, cancellous bone of inferior third of humeral head manually impacted in an inferior-to-superior direction with flat tip of a 0.64 cm rod to simulate bone loss or comminution  Special testing system used to perform all test.  **Cyclic Abduction Test:** 30^o^-120^o^ at 0.1 Hz (joint compressive load 0.2-0.5 body weight) for 250 cycles  **Cyclic Torsion Test:** 0.1-2.1 Nm cyclic external torque at 0.5 Hz with a 10 N constant compressive load for 250 cycles  **Static Rotation Test:** Load at 1°/s until failure  **Failure:** For rotation tests, 30°rotational displacement | Recorded humeral head, shaft and greater tuberosity interfragmentary motion (using optoelectronic camera system)    **Cyclic Abduction Test:** Determined head translation after cycling  **Torsion Tests:** Determined head rotation after cycling and torsional stiffness and load to failure |
| **TYPE 3 & 4 LOADING** | | | | |
| Lever et al., 2008 (Lever et al., 2008) | AO low contact dynamic compression plate (Synthes)  Modified AO low contact dynamic compression plate (Synthes) into a “blade plate”  AO dynamic compression blade plate (Synthes)  AO, T-plate (Synthes)  Modified AO cloverleaf plate (Synthes) | Human cadaver humeri | **Fracture:** Two-part fracture, transverse surgical neck osteotomy 10 mm distal to inferomedial margin of articular surface of humeral head  Initial non-destructive testing of humeri within elastic region to determine their stiffness  **20^o^ Abduction and Flexion Test:** Humeri fixed at 20^o^ of abduction and forward flexion, 100 N applied at superior end  **90^o^ Abduction and Flexion Test:** Humeri fixed at 90^o^ of flexion, 100 N applied on lesser tuberosity, in posterior direction, then humeri fixed at 90^o^ of abduction, 100 N applied on centre of greater tuberosity, in medial direction  **Failure:** N/A | Recorded force and displacement, determined stiffness and normalised stiffness |
| **TYPE 1, 2 AND 3 LOADING** | | | | |
| Lill et al., 2003 | Humerus T-plate (Synthes)  Locking compression plate (Mathys, Bettlach)  Cross-screw osteosynthesis  Humerus nail with spiral blade (Synthes)  Synclaw humerus nail | Human cadaver humeri | **Fracture:** Two-part fracture, transverse osteotomy with 5 mm gap  **Static Axial Compression Test:** 0.5 mm axial compression  **Static Torsion Test:** 4^o^ axial rotation  **Static Varus Bending Test:** Latero-medial (varus) bending by applying 4 mm compressive displacement  **Cyclic Test:** Varus bending for 1000 cycles  **Failure:** N/A | Recorded relative movements at fracture gap using optical measuring system  **Static Tests:** Recorded force and displacement, determined compressive, torsional and bending stiffness  **Cyclic Test:** Recorded moment and number of cycles, determined maximum load during first cycle, load at 300 cycles, load reduction (%) due to migration or loosening throughout the 1000 cycles and the slope of the load-cycles curve between cycles 700 and 1000 |
| Duda et al., 2007 | ButtonFix PEEK plate (Synthes)  Humerus Block (Synthes) | Human cadaver humeri | Same as above (Lill et al., 2003) | Same as above (Lill et al., 2003) |
| **TYPE 1, 2 AND 4 LOADING** | | | | |
| Lescheid et al., 2010 | AxSOS plate (Stryker) | Synthetic humeri (Sawbones 3^rd^ generation, composite) | **Fracture:** Two-part fracture, transverse wedge osteotomies in all specimens to simulate bone loss about medial calcar region at 0^o^, 10^o^, and 20^o^ while second osteotomy created for half of specimens to remove a 10 mm segmental defect  **Static Axial Test:** Axial compression at 5 mm/min with 50 N preload until 0.2 mm deflection  **Static Torsion Test:** Internal rotation at 1 o/s and 0 Nm pre-torque, until 1^o^  **Static Shear Test (LT4):** Humeri fixed at 20^o^ of abduction and same procedure as static axial test until 0.75 mm maximum displacement  **Static Shear Strength Test (LT4):** Vertical force to generate shear compression until catastrophic failure of the implant or the humerus at 5 mm/min with 50 N preload  **Failure:** N/A | **Static Axial Test:** Recorded force and displacement, determined axial stiffness  **Static Torsion Test:** Recorded torque and angle, determined torsional stiffness  **Static Shear Test (LT4):** Recorded force and displacement, determined shear stiffness  **Static Shear Strength Test (LT4):** Determined shear load to failure |
| Katthagen et al., 2014 | PHILOS plate (Synthes) | Human cadaver humeri | **Fracture:** Two-part fracture with comminuted medial cortex and bone loss in medial calcar region, transverse wedge osteotomy with 10 mm gap at surgical neck region  **Static Torsion Test**: Torsional load to humeral head at 0.1 o/s until ±3.5 Nm  **Static Axial and Shear Test (LT4)**: Vertical load on humeral head (axial, 20° abduction, 20° adduction) at 0.1 mm/s until 200 N  **Cyclic Axial Test**: 50-250 N sinusoidal axial compression load at 1 Hz for 5000 cycles  **Static Load to failure:** Vertical load at 0.1 mm/s until failure  **Failure:** Implant failure, fracture gap closure (touching of medial cortices) or fracture around humeral head or shaft | Recorded 3D fracture gap displacement using an ultrasound based device  **Static Torsion Test**: Determined torsional stiffness  **Static Axial and Shear Test (LT4)**: Determined axial and shear (bending) stiffness  **Cyclic Axial Test**: Determined mean displacement for 5000 cycle testing period and maximum displacement after 5, 50, 100, 2500 and 5000 cycles  **Static Load to failure:** Determined load to failure |
| Katthagen et al., 2016 | PEEKPower plate (Arthrex) | Human cadaver humeri | First half identical to above (Katthagen et al.) but additionally performed screw perforation test  **Static Screw Perforation Test:** Load applied at 0.05 mm/s, pressing humeral head articular fragment against screw tips until visual observation of screw perforation with exposed screw tip and/or cartilage tear | Identical to above (Katthagen et al.), additionally determined load until first screw perforation |
| Bai et al., 2014 | PHILOS plate (Synthes) | Human cadaver humeri | **Fracture:** 5 mm transverse wedge osteotomy on one set of specimen and 20^o^ humeral head collapse simulated on another set  **Static Axial and Shear Test (LT4):** Vertical load at 5 mm/min with 50 N preload and 500 N maximum load until 5 mm maximum compression  **Static Torsion Test:** ±5^o^ load at 5 o/min  **Cyclic Axial Test:** 50-300 N load for 5000 cycles until 5 mm maximum deflection  **Failure:** N/A | **Static Tests:** Recorded force and displacement, determined stiffness  **Cyclic Test:** Recorded maximum and minimum displacements for each cycle, determined displacements in 1, 10, 200, 400, 600, 800, 1000, 2000, 3000, 4000, and 5000 cycles |
| Zhang et al., 2014 | Humeral locking plate (Double Medical) | Synthetic humeri (Orthobone) | **Fracture:** Two-part fracture of surgical neck  **Static Axial Test:** Vertical displacement at 5 mm/min with 50 N preload until 0.5 mm maximum deflection  **Static Shear Test (LT4):** Humerus fixed at 20^o^ of abduction, same test procedure as static axial testing with 1.0 mm maximum displacement  **Static Torsion Test:** Rotation at 12 o/min with 0 Nm preload, until 5^o^ maximum angle  **Static Shear Failure Test (LT4):** Humerus fixed at 20^o^ of abduction, loading at 5 mm/min with 50 N preload until failure  **Failure:** Catastrophic implant failure | **Static Axial and Shear Tests(LT4):** Recorded force and displacement, determined stiffness and maximum load  **Static Torsion Test:** Recorded force and rotation, determined torsional stiffness and maximum torque  **Static Shear Failure Test (LT4):** Determined shear failure load |
| **TYPE 2, 3 AND 4 LOADING** | | | | |
| Sanders et al., 2007 | Humerus locking plate (Synthes)  Polaris intramedullary nail (Accumed) | Human cadaver humeri | **Fracture:** Three-part fracture, intertubercular groove and surgical neck 1 cm distal to articular surface, separating head, greater tuberosity, and shaft  **Static Eccentric Loading (LT4):** 0-80 N load 2.5 cm from shaft axis at 10 N/s  **Force Vector Test (LT4):** 80 N force vector 30° posteromedial to anteroposterior plane at 10 N/s  **Static Torsion Test:** 0-8^o^ rotation at 1°/s  **Cyclic Bending Test:** 500 N (amplitude ± 100 N) load at 10 Hz for 16,000 cycles  **Final Static Test**: Load at 100 N/s until failure  **Failure:** Acute decrease in load indicating loss of construct’s mechanical integrity | **Static Eccentric (LT4) and Force Vector Loading:** Recorded force and displacement, determined stiffness  **Static Torsion Test:** Recorded torque and angular displacement, determined rotational stiffness  **Cyclic Bending Test:** Recorded number of cycles, determined peak load  **Final Static Test:** Determined load to failure |
| **HUMERUS-TENDON LOADING** | | | | |
| Walsh et al., 2006 | Locking compression plate (Synthes)  Cloverleaf plate (Synthes) | Human cadaver humeri | **Fracture:** Two-part fracture, 7 mm osteotomy at level of surgical neck  Simulated 30^o^ abduction of glenohumeral joint using custom-made testing mechanism to pull supraspinatus, subscapularis and infraspinatus at 0.5 mm/s  **Failure:** N/A | Recorded force and displacement, determined maximum load prior to failure |
| Voigt et al., 2011 | PHILOS plate (Synthes) | Human cadaver humeri | **Fracture:** Closed-wedge osteotomy, with first subcapital osteotomy at level of surgical neck 5 cm distal to top of head from medial to lateral, and second osteotomy at target deformity angle (20^o^ or 45^o^) from inferior–medial to superior–lateral  Simulated rotator cuff tension (glenohumeral elevation) using robot-assisted shoulder simu­lator (RASS, KUKA Robotics) to apply 66, 26 and 61 N to subscapularis, supraspinatus and infraspinatus/teres minor, respectively  **Failure:** N/A | Determined deltoid forces necessary to elevate the arm in set positions, supraspinatus efficiency and ratio of deltoid force to arm elevation angle in different phases of elevation |
| Voigt et al., 2009 | PHILOS plate  Humeral suture plate (Arthrex) | Human cadaver humeri | **Fracture:** Three-part fracture, greater tuberosity osteotomy 5 cm distal to the top of the humeral head and ventral directly lateral to bicipital groove  Simulated rotator cuff tension (glenohumeral elevation) using robot-assisted shoulder simu­lator (RASS, KUKA Robotics) with same muscles and pulling force as above (Voigt et al., 2011)  Also simulated:   1. 120 N Axial loading at 0° glenohumeral abduction by loading all rotator cuff muscles to their physiological tensions in neutral joint position 2. Axial loading at 60° glenohumeral abduction by loading all rotator cuff muscles to their physiological tensions in neutral joint position 3. Internal rotation at 0° abduction by loading subscapularis at its physiological tension and supraspinatus and infraspinatus/teres minor to 10 N to simulate co-contraction 4. External rotation at 0° abduction by loading infraspinatus/teres minor to their physiological tensions and subscapularis and supraspinatus to 10 N   **Failure:** N/A | Recorded interfragmentary motion using ultrasound based 3D motion analysis system and measured rotator cuff strain using an optical system |
| Rose et al., 2010 | S3 plate (Depuy)  Proximal humerus locking compression plate (Synthes) | Human cadaver humeri | **Fracture:** Three-part fracture, 10 mm bone segment removed first and an additional osteotomy through intertubercular groove  Custom-made testing frame used with biaxial servohydraulic testing machine  SSP pull simulated by producing 10^o^-60^o^ cyclic abduction using 40-200 N sinusoidal load at 0.33 Hz for 5000 cycles or until failure  **Failure:** Gross failure or loss of fixation | Recorded relative interfragmentary displacement between head and shaft and between head and greater tuberosity fragment using an optical motion tracking system, determined calcar and tuberosity displacement location and head rotation after 5000 cycles |
| Sinatra et al., 2014 | Proximal humerus locking plate (Synthes) | Human cadaver humeri | **Fracture:** Two-part fracture, first 15 mm resection osteotomy, second osteotomy 50 mm distal to plate’s distal end  Used custom-made shoulder testing setup connected to a material testing machine. 50-100^o^ shoulder abduction simulated by applying 125-200 N cyclic tensile forces to supraspinatus, infraspinatus, subscapularus, and teres minor tendons at 0.5 Hz and 30 o/s for 400 cycles, while lifting 5 lbs (~2.27 kg) to simulate arm weight  **Failure:** Ultimate tensile strength of construct | Recorded fracture gap distance using video recorder, determined intercyclic motion (change in fracture gap within a single loading cycle at both 125 and 200 N) at 100, 200, 300, and 400 cycles  Also determined load to failure and residual fracture gap deformation (difference in fracture distance at beginning and end of 400 cycles) |
| Arvesen et al., 2016 | Proximal humerus locking plate (Synthes) | Human cadaver humeri | **Fracture:** Three-part fracture, first a 15-mm resection osteotomy at surgical neck and then a greater tuberosity osteotomy at the junction of the articular margin and extending distally along lateral aspect of bicipital groove  Used custom-made shoulder testing setup which allowed single plane abduction by tensile loading of supraspinatus, subscapularis, and teres minor tendons. To induce 35-65^o^ active glenohumeral abduction, cyclic load applied to rotator cuff tendons (0.5 Hz at 30^o^/s) while lifting 5 lbs to simulate arm weight. Peak loads increased from 125 to 200 N and test stopped after 400 cycles. Finally, 1000 N applied at 35^o^ glenohumeral abduction.    **Failure**: Disruption of the pulley–tendon interface, hardware failure, or complete displacement of the fracture fragments | High-definition video recorder used to record fracture gap distance of  surgical neck and greater trochanter during abduction  At 100, 200, 300, and 400 cycles, intercyclic motion recorded as change in fracture gap. Residual fracture gap deformation recorded as difference in fracture distance at the beginning and end of the 400 cycles.    For 1000 N load test, measured fracture gap displacements at greater tuberosity and surgical neck |
| Brunner et al., 2012 | Humerus Block: New Generation with locked telescoping pins (Synthes) | Human cadaver humeri | **Fracture:** Two-part fracture, first wedge osteotomy with 0.5 cm lateral fracture gap 1 cm below medial border of anatomical neck, medial hinge later resected to create a 0.5 cm fracture gap  Shoulder test bench used with pneumatic muscles attached to original insertions of supraspinatus, deltoideus, teres major and pectoralis major muscle  15^o^-45^o^ abduction and adduction simulated by loading for 500 cycles. In each cycle, deltoideus and supraspinatus pneumatic muscle loads increased stepwise until 45^o^ humerus abduction, then teres major and pectoralis major pneumatic muscle loads increased stepwise until 15^o^ arm abduction  **Failure:** N/A | Recorded interfragmentry motion using 3D motion analysis system, degree of abduction using inclinometer and muscle forces, determined implant migration by performing X-rays before testing and after every 500 cycles  Determined mean maximal sintering and mean per cycle movement of head  Determined mean maximum varus tilt (angulation) and per cycle varus tilt (angulation) of head |
| Kathrein et al., 2013 | PHILOS plate (Synthes) | Human cadaver humeri | **Fracture:** Two-part fractures, wedge osteotomy model 1 cm below anatomical neck with 1 cm wedge at base, gap osteotomy model later simulated by removing medial hinge  Custom-made testing setup used with pneumatic muscles simulating supraspinatus, deltoideus, pectoralis major and teres major  Simulated 15°-45° abduction and adduction under each fracture model for 500 cycles  **Failure:** N/A | Recorded fracture gap movement using ultrasound based motion analysis system, determined varus impaction and per cycle relative motion of humeral head and plate |
| Da Graca et al., 2013 | Dynamic compression plate (Synthes)  Transosseous sutures fixations with cortical screws and Kirschner wires | Synthetic humeri (Nacional Ossos plastic humeri, aluminium scapula) | **Fracture:** Four-part fracture, with fragments: head dome, lesser tuberosity, greater tuberosity and diaphysis  Custom-made testing setup based on universal testing machine used with calf leather straps to simulate infraspinatus, supraspinatus, subscapularis and axillary capsular recess  Simulated resisted shoulder abduction for 30 s with 5 N preload force, then loaded at 20 mm/min until failure, all with fixed scapula  Simulated resisted shoulder internal for 30 s with 30 N preload force, then loaded at 20 mm/min until failure, all with fixed scapula and humeral shaft fixed in rotary accessory  **Failure**: Sudden drop in applied load | Recorded force and displacement, determined relative bending and torsional rigidity |
| Osterhoff et al., 2011 | PHILOS plate (Synthes) | Synthetic humeri (Synbone composite) | **Fracture:** Two-part fracture, simulated metaphyseal comminution zone by removing 10 mm section at greater tuberosity base  Custom-made shoulder testing device connected to universal testing machine using artificial tendons  Simulated 45^o^ to 60^o^ abduction movement by applying 50-125 N to supraspinatus and deltoideus and 25 N to infraspinatus/teres minor and subscapularis at 300 mm/min for 400 cycles while lifting 3.75 kg arm weight to achieve 5 o/s humeral abduction speed  **Failure:** N/A | Recorded fragment gap distance, determined intercyclic motion (fragment gap amplitude within 1 loading cycle) determined at 100, 200, 300, and 400 cycles  Determined fragment migration (change in fragment gap distance during cyclic testing in loaded condition) and residual plastic deformation (difference in fragment gap distance before and after 400 loading cycles in unloaded condition) |
| Clavert et al., 2016 | PHILOS plate (Synthes)  Aequalis nail | Synthetic humeri (Sawbones 4^th^ generation) | **Fracture:** Four-part fracture, humeral neck osteotomy performed as well as intertuberosity osteotomy 8 mm lateral to the bicipital groove  Custom-made testing setup connected to mechanical testing machine used with polyethylene rope glued to superior and lateral greater tuberosity aspects  Simulated 0° glenohumeral abduction and neutral rotation relative to scapula plane or 90° of abduction in scapula plane by translating proximal humerus inferiorly at 50 mm/s with 10 N preload  **Failure:** N/A | Recorded force, displacement and strain applied on greater tuberosity, determined stiffness and load to failure |
